# Characterising spinal cerebrospinal fluid flow in the pig with phase-contrast magnetic resonance imaging

**DOI:** 10.1186/s12987-022-00401-4

**Published:** 2023-01-18

**Authors:** Madeleine Amy Bessen, Christine Diana Gayen, Ryan David Quarrington, Angela Catherine Walls, Anna Victoria Leonard, Vartan Kurtcuoglu, Claire Frances Jones

**Affiliations:** 1 Adelaide Spinal Research Group and Centre for Orthopaedics and Trauma Research, Adelaide Medical School, The University of Adelaide, Level 7, Adelaide Health and Medical Sciences Building, The University of Adelaide, North Terrace, Adelaide, SA 5005 Australia; 2 Translational Neuropathology Laboratory, School of Biomedicine, The University of Adelaide, Level 2, Helen Mayo North Building, The University of Adelaide, Frome Road, Adelaide, SA 5005 Australia; 3 Clinical and Research Imaging Centre, South Australian Health and Medical Research Institute, National Imaging Facility, Northern Pod, SAHMRI, North Terrace, Adelaide, SA 5000 Australia; 4 Institute of Physiology, University of Zurich, Winterthurerstrasse 190, 8057 Zurich, Switzerland; 5 Zurich Center for Integrative Human Physiology, University of Zurich, Winterthurerstrasse 190, 8057 Zurich, Switzerland; 6 Neuroscience Center Zurich, University of Zurich, Winterthurerstrasse 190, 8057 Zurich, Switzerland; 7 School of Electrical and Mechanical Engineering, The University of Adelaide, North Terrace, Adelaide, SA 5005 Australia; 8 Department of Orthopaedics, Royal Adelaide Hospital, Adelaide, SA 5000 Australia

**Keywords:** Cerebrospinal fluid, Pulsatile, Spine, Flow, Velocity, Phase-contrast magnetic resonance imaging, Pig

## Abstract

**Background:**

Detecting changes in pulsatile cerebrospinal fluid (CSF) flow may assist clinical management decisions, but spinal CSF flow is relatively understudied. Traumatic spinal cord injuries (SCI) often cause spinal cord swelling and subarachnoid space (SAS) obstruction, potentially causing pulsatile CSF flow changes. Pigs are emerging as a favoured large animal SCI model; therefore, the aim of this study was to characterise CSF flow along the healthy pig spine.

**Methods:**

Phase-contrast magnetic resonance images (PC-MRI), retrospectively cardiac gated, were acquired for fourteen laterally recumbent, anaesthetised and ventilated, female domestic pigs (22–29 kg). Axial images were obtained at C2/C3, T8/T9, T11/T12 and L1/L2. Dorsal and ventral SAS regions of interest (ROI) were manually segmented. CSF flow and velocity were determined throughout a cardiac cycle. Linear mixed-effects models, with post-hoc comparisons, were used to identify differences in peak systolic/diastolic flow, and maximum velocity (cranial/caudal), across spinal levels and dorsal/ventral SAS. Velocity wave speed from C2/C3 to L1/L2 was calculated.

**Results:**

PC-MRI data were obtained for 11/14 animals. Pulsatile CSF flow was observed at all spinal levels. Peak systolic flow was greater at C2/C3 (dorsal: − 0.32 ± 0.14 mL/s, ventral: − 0.15 ± 0.13 mL/s) than T8/T9 dorsally (− 0.04 ± 0.03 mL/s; p < 0.001), but not different ventrally (− 0.08 ± 0.08 mL/s; p = 0.275), and no difference between thoracolumbar levels (p > 0.05). Peak diastolic flow was greater at C2/C3 (0.29 ± 0.08 mL/s) compared to T8/T9 (0.03 ± 0.03 mL/s, p < 0.001) dorsally, but not different ventrally (p = 1.000). Cranial and caudal maximum velocity at C2/C3 were greater than thoracolumbar levels dorsally (p < 0.001), and T8/T9 and L1/L2 ventrally (p = 0.022). Diastolic velocity wave speed was 1.41 ± 0.39 m/s dorsally and 1.22 ± 0.21 m/s ventrally, and systolic velocity wave speed was 1.02 ± 0.25 m/s dorsally and 0.91 ± 0.22 m/s ventrally.

**Conclusions:**

In anaesthetised and ventilated domestic pigs, spinal CSF has lower pulsatile flow and slower velocity wave propagation, compared to humans. This study provides baseline CSF flow at spinal levels relevant for future SCI research in this animal model.

**Supplementary Information:**

The online version contains supplementary material available at 10.1186/s12987-022-00401-4.

## Background

Cerebrospinal fluid (CSF) cushions the brain and spinal cord from mechanical injury, acts as a critical transport medium for nutrients and metabolites to maintain a homeostatic environment for neuronal cells, and provides a drainage system for interstitial fluid from the central nervous system (CNS) [1]. Although the CSF system remains relatively poorly understood, the diagnostic [2–6] and therapeutic [7, 8] potential of CSF dynamics have become increasingly apparent. Free-flowing CSF is vital to brain and spinal cord health, and changes to CSF flow may adversely affect fluid balance and metabolism [9–11].

CSF flow in humans is predominantly oscillatory, with cranial and caudal pulsations along the spinal cord axis synchronous with the cardiac and respiratory cycles [12]. CSF pulsations can be measured with phase-contrast magnetic resonance imaging (PC-MRI). A bipolar gradient is applied in the pulse sequence to produce a phase shift, which is directly proportional to proton velocity in the flow-encoding direction [13], and is gated with cardiac activity to produce velocity data as a function of the cardiac cycle. PC-MRI studies of CSF flow in the cranium and spinal canal have been fundamental in improving understanding of CSF dynamics in normal and disease states [14–18]. CSF flow in the cerebral aqueduct and at the cranio-cervical junction are used to assist in the diagnosis of hydrocephalus [2–4, 19] and Chiari malformation [5, 6, 20, 21]. Although CSF dynamics in the spinal column may provide similar diagnostic capabilities, the spinal region remains relatively understudied. Several studies have characterised normal CSF dynamics along the spine in healthy humans [22–25] and non-human primates (NHP) [26]. Other studies have found that obstructions to the intrathecal space may cause changes to CSF dynamics in the context of cervical myelopathy [27–29] and in neurogenic claudication in lumbar spinal stenosis [30].

Traumatic spinal cord injury (SCI) may alter CSF flow due to the swollen spinal cord obstructing the spinal subarachnoid space (SAS) [31, 32]. One study demonstrated CSF velocity changes at the level of injury in cervical SCI patients up to two years after injury [33], and another study of post-traumatic syringomyelia identified altered temporal features and lower peak velocities at the syrinx level in patients compared to healthy humans [34]. Pigs are emerging as a favoured large animal model for SCI research [35]. The more human-like size of the porcine spinal column [36], spinal cord, and intrathecal space [37, 38] compared to rodents, provides the opportunity to study CSF dynamics using clinical imaging modalities and in-dwelling pressure sensors. The anterior–posterior diameter of the spinal canal at T10 is approximately 20 mm in humans, 10 mm in 40 kg domestic pigs [36], and 2.7–3.3 mm at T12–L1 in rats [39]. It is important to examine normal spinal CSF flow in the pig to provide a benchmark for future investigations of SCI. The aim of this study was to characterise normal CSF flow along the pig spine, examining two locations proximal, and two locations distal, to thoracic level T10, which is the standard spinal level for pre-clinical contusion models of SCI [40].

## Methods

### Animal ethics

This project was approved by the South Australian Health and Medical Research Institute Animal Ethics Committee (SAM 243 and SAM-22-031) and conducted in accordance with the Australian National Health and Medical Research Council Code of Care and Use of Animals for Scientific Purposes [41].

### Animals

Magnetic resonance images (MRI) of fourteen healthy, female, Large White Landrace cross pigs (22–29 kg at imaging) were obtained. The animals were acclimatised in a purpose-built facility for 7–10 days prior to imaging. During this time they were provided enrichment toys, access to water, twice daily food rations, and administered once daily prophylactic antibiotics (Trimidine™ 0.15gm/10 kg; Sulfadimidine 430 mg, Trimethoprim 86 mg). After imaging was completed, animals were recovered from anaesthetic, and maintained for further approved procedures on the same ethics protocol that are not reported herein, after which they were humanely killed.

### Anaesthetic protocol

The animals were fasted overnight prior to anaesthesia and pre-medicated with medetomidine (0.02 mg/kg) and ketamine (7.5 mg/kg). Anaesthesia was induced using ketamine (11 mg/kg) and propofol (2 mg/kg), maintained with an intravenous protocol of ketamine (5.0–8.0 mg/kg/hr), propofol (2.0–6.0 mg/kg/hr), and fentanyl (8.0–15.0 ug/kg/hr) and titrated as necessary towards the end of the imaging session. A propofol bolus (2.0 mg/kg) was administered when vital signs and/or physical indications suggested lightening of anaesthesia. Anaesthetics were administered via a venous catheter that was placed in the external jugular vein via surgical cut-down immediately prior to the MRI. The animals were intubated, ventilated at 17–21 breaths/min with 270—400 mL/min of oxygen, and heart rate, oxygen saturation and end-tidal carbon dioxide were monitored.

### Magnetic resonance imaging

MRIs were performed on a 3T scanner (Siemens, Magnetom Skyra, Germany). Animals were placed in left lateral recumbency with two 18-channel body coils (Siemens, Germany) wrapped over their torso and neck. T2-weighted TSE (turbo spin echo) images of the cervical/thoracic and thoracic/lumbar spinal regions were acquired with the following parameters: sagittal slice thickness 4 mm, in-plane resolution 1.302 × 1.302 mm, repetition time (TR) 3500 ms, echo time (TE) 98 ms, 2 averages, 146° flip angle and 384 × 384 acquisition matrix. These images were used during scanning to prescribe the PC-MRI axial locations and orientations, and post-processed to measure spinal length (see below).

### Phase-contrast magnetic resonance imaging acquisition

**Fig. 1:**
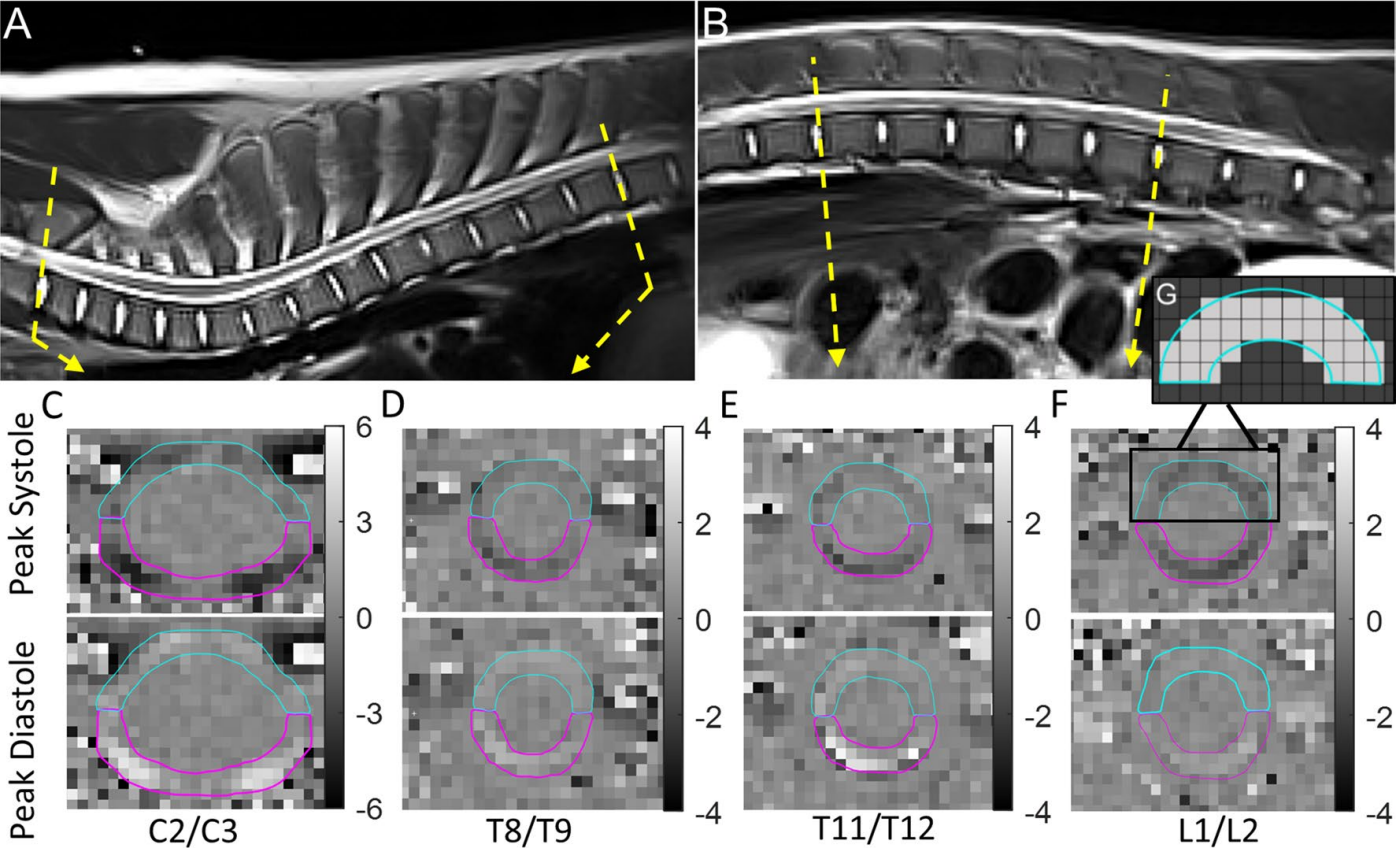
Sagittal T2 TSE MRI of the **A** cervical and thoracic spine and **B** thoracic and lumbar spine from one representative animal with labelled spinal levels and planes of the phase-contrast MRI (PC-MRI) images. **C**–**F** Representative axial PC-MRI (phase) at peak systolic (caudal) and at peak diastolic (cranial) flow for each spinal level with scale bar (velocity; cm/s). **G** A schematic demonstrating which pixels are assigned within the ROI. Blue is the ventral SAS ROI; pink is the dorsal SAS ROI. *TSE* turbo spin echo, *MRI* magnetic resonance imaging, *ROI* region of interest, *SAS* subarachnoid space

PC-MRI acquisition was performed with retrospective cardiac gating using a pulse oximeter attached to the tail. At spinal levels C2/C3, T8/T9, T11/T12 and L1/L2, a single axial slice orthogonal to the spinal cord in the sagittal and coronal planes, aligned with the centre of the adjacent intervertebral disc, was acquired (Fig. 1A-G). Cranial flow was positively encoded (white pixels) and the number of acquired cardiac phases ranged from 14 to 30, selected according to the target R-R interval. Acquisition duration per spinal level was between 3 and 9 min. Minimum TR/TE was selected within the pulse sequence. PC-MRI sequence parameters for the first ten animals included: field of view, 140 × 140 mm; TR, 46.39–59.84 ms; TE, 9.41 ms; in-plane resolution, 0.5469 × 0.5469 mm; slice thickness 5 mm; anterior–posterior phase-encoding direction; 15° flip angle. Encoding velocities (V_ENC_) of 4 cm/s and 6 cm/s were selected during a pilot study and run consecutively at each spinal level. One animal was also scanned with an additional V_ENC_ of 2 cm/s at each spinal level and the TR and TE changed to 55.61–69.02 ms and 12.41 ms, respectively. Following completion of the first ten animals, aliasing was detected in some animals at C2/C3 in the V_ENC_ = 6 cm/s scans; therefore, for the last four animals (P011-P014), V_ENC_ of 6 cm/s, 8 cm/s and 10 cm/s were used at that level. Because of this increase in V_ENC_, TR and TE changed to 59.84 ms and 8.04 ms, respectively, at C2/C3; all other parameters remained the same.

### Phase-contrast magnetic resonance imaging post-processing

Post-processing of CSF flow images was undertaken by a single investigator (MAB) using Segment software (Version 3.2, Medviso, Lund, Sweden) [42]. At each spinal level, regions of interest (ROI) were manually defined at the border of the CSF flow signal in the SAS, on the image that displayed the greatest contrast between CSF flow signal and the surrounding tissues. ROIs were defined separately in the ventral and dorsal SAS (referred to as the SAS region), because of its potential relevance to spinal pathologies [11, 33] and because in some animals flow signal was observed in one region and not the other. The dorsal region was split into two ROIs when distinct compartments were observed, to ensure only pixels with flow signal were selected (Additional file 1: Fig. S1). In the Segment software, pixel selection for flow estimation included only those pixels with their centroid inside the contour of the ROI (Fig. 1G). Motion of the spinal canal was not observed on the re-phased [43] (anatomical) image at any spinal level, and the ROIs were propagated to each temporal image. To assess intra-rater reliability, repeat ROIs were completed two months later by the same investigator, on ten randomly selected PC-MRIs, across all spinal levels and animals.

Eddy current offset correction with time-dependent linear compensation was applied by iteratively adjusting the threshold in Segment software until the sum of the net flow of the two ROIs was as close to zero as possible. The resulting residual net flow was determined as a percentage of the total stroke volume (mL/cycle) of both ROIs. This was performed on all scans that captured the full systolic and diastolic flux. Residual net flow in all animals was less than 9% (2.85 ± 2.47%; Additional file 1: Table S1). The mean offset magnitude applied at C2/C3 was 0.05 ± 0.05 mL/s, at T8/T9 was 0.03 ± 0.02 mL/s, at T11/T12 was 0.02 ± 0.02 mL/s, and L1/L2 was 0.03 ± 0.04 mL/s. Each ROI was inspected for aliasing artefact (i.e., maximum velocity exceeding the acquisition V_ENC_), and aliasing was corrected with phase-unwrapping which was confirmed visually by examining the pixel-by-pixel velocity in each image. This was performed on 8 animals at C2/C3, 1 animal at T11/T12, and 2 animals at L1/L2. At most 5 consecutive cardiac phases were corrected (mean consecutive corrected phases: 2 ± 1), and no more than 14% of the total number of pixels in both ROIs were corrected at C2/C3, 2% at T11/T12, and 9% at L1/L2. The following parameters were derived: CSF velocity (cm/s) in each ROI for each cardiac phase acquired; CSF flow (mL/s) in each ROI for each cardiac phase acquired; stroke volume, defined as the sum of the caudal flow volume and the cranial flow volume in one cardiac cycle (mL/cycle); maximum cranial and caudal velocity (single pixel; cm/s) in each SAS region, over the cardiac cycle.

These data were further processed using a custom MATLAB program (Version R2020a, Mathworks Inc, Natick, MA). For dorsal SAS with two ROIs, flow data were summed, and mean velocity were averaged, to provide a single dorsal value for each outcome. Flow and mean velocity versus time data for each cycle were interpolated to 100 points using a cubic spline (“pchip” MATLAB function to preserve minima and maxima), and expressed as a percentage of the cardiac cycle to normalise these data by heart rate. The following parameters were derived: peak systolic (caudal direction) and peak diastolic (cranial direction) flow; and, mean velocity, defined as the mean velocity of all pixels within the ROI, which was used to calculate the time to peak mean velocity (systolic and diastolic; from t = 0 in the cardiac cycle).

### Velocity wave propagation speed

CSF velocity wave speed (VWS) [44] was estimated for each animal as the distance between the C2/C3 and L1/L2 spinal levels divided by the time taken for the velocity wave (peak systolic, peak diastolic) to travel that distance. The distance from C2/C3 to the L1/L2 intervertebral disc was measured across both T2 FSE scans (summed distance from C2/C3 to T11/T12, and T11/T12 to L1/L2) with a spline placed along the centre of the spinal cord on a sagittal view, using Materialise Mimics software (Version 22.0, Materialise, NV). For the diastolic velocity wave, the difference between time to peak mean diastolic velocity at C2/C3 and L1/L2 was calculated. For the systolic velocity wave calculation, time to peak mean systolic velocity at L1/L2 was adjusted: cardiac cycle duration minus time to peak mean systolic velocity at C2/C3 was added to the time to peak systolic velocity at L1/L2. Systolic velocity wave speed was calculated as the difference between time to peak mean systolic velocity at C2/C3 and the adjusted L1/L2 value.

### Statistical analyses

All statistical analyses were performed with SPSS (version 26, IBM Corporation, Armonk, NY). Intra-rater reliability of the ROI area, and the corresponding peak diastolic and systolic flow, was determined using intraclass correlation coefficients (ICC) of the absolute agreement between the repeated ROI segmentations. ICC values > 0.75 and < 0.9 were defined as good reliability, and values > 0.9 as excellent reliability [45]. Normality and homogeneity of variance were assessed for peak systolic flow, peak diastolic flow, and maximum cranial and caudal CSF velocity data using Shapiro–Wilk and Levene’s tests. Linear mixed-effects models (LMMs) were developed to identify if there was an effect of the spinal level, SAS region (ventral and dorsal ROI), the spinal level*SAS region interaction, on peak systolic and diastolic flow, and maximum cranial and caudal CSF velocity. LMMs initially also included a fixed effect of temporal resolution (cardiac cycle duration divided by the number of cardiac phases acquired); however, it was not associated with any outcome measures, and was subsequently removed from the models. Post-hoc pairwise comparisons, with Bonferroni correction, were performed for LMMs with significant interactions (significance level of α = 0.05). Experimental data is presented as mean ± standard deviation (SD) in text, with the p-values corresponding to the LMMs, and complete LMM outcomes are available in the supplementary materials.

## Results

Complete CSF flow data were obtained for 11 of 14 animals. CSF flow was not detected at any spinal level in 3 animals (P005, P011, and P013). In one animal (P003), CSF flow was not detected at T8/T9 and T11/T12. CSF flow was not detected in the dorsal SAS at T8/T9 in 4 of 11 animals (Additional file 1: Table S2). When CSF flow was not detected and a lower V_ENC_ was not scanned, the spinal level and/or SAS region was excluded from all analyses and plots; zero flow could not be confirmed since it is possible that there was flow below a detectable threshold determined by the amount of phase noise present. Two animals (P004 and P012) had abnormal cardiac gating and optimised eddy current corrections could not be performed since the full systolic and diastolic flux was not captured (Additional file 1: Figure S2). Data for these animals were excluded from temporal analyses and plots, but included in peak flow values since the diastolic and systolic extremes were maintained and were within, or marginally outside the range of the remaining data (Additional file 1: Table S3). The mean heart rate for the duration of the scans was 101 ± 27 bpm (Additional file 1: Table S4).

For all levels except C2/C3, the dorsal ROIs were only slightly smaller in area than the ventral ROIs (Table 1). ROI area and associated peak diastolic and systolic flow, in the dorsal SAS and ventral SAS, demonstrated excellent intra-rater reliability and almost perfect agreement between the two measurements (Table 1).

**Table 1:** ROI area (mean ± SD) for all animals with detected flow signal (n = 11), and intraclass correlation coefficients (ICC) for ROIs manually defined two months apart by the same investigator using a sample of ten PC-MRIs

	Dorsal SAS	Ventral SAS
ROI area for C2/C3 (mm^2^)	14.9 ± 4.9	10.1 ± 5.0
ROI area for T8/T9 (mm^2^)	10.0 ± 4.2	10.3 ± 3.6
ROI area for T11/T12 (mm^2^)	10.6 ± 2.3	10.9 ± 4.3
ROI area for L1/L2 (mm^2^)	10.8 ± 4.1	12.9 ± 3.2
ICC: ROI area	0.899	0.901
ICC: Peak diastolic flow	0.953	0.944
ICC: Peak systolic flow	0.977	0.944

*ROI* region of interest, *SAS* subarachnoid space, *PC-MRI* phase contrast magnetic resonance imaging

### CSF flow

**Fig. 2:**
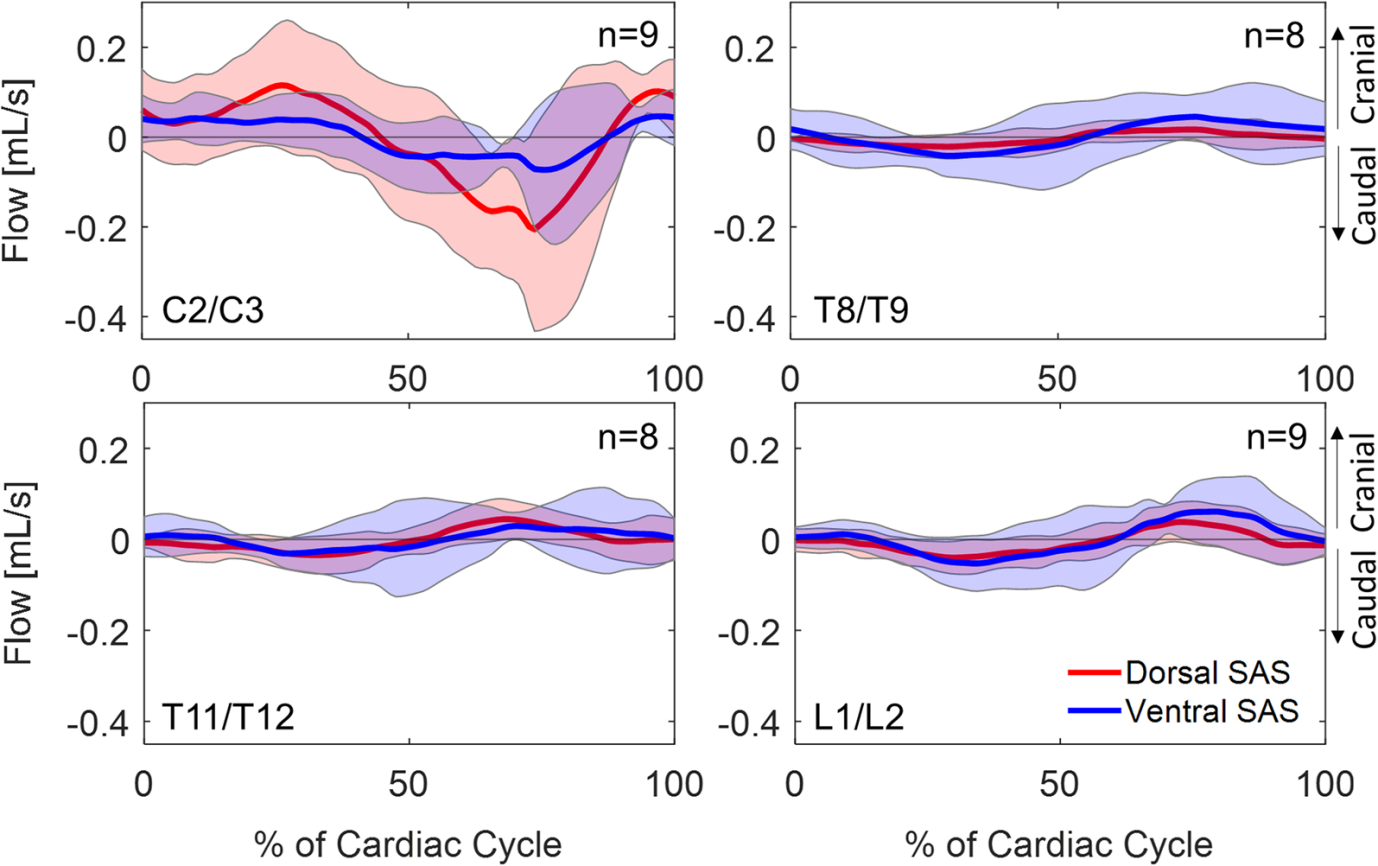
CSF flow over one cardiac cycle from PC-MRI data (n = 9). The red line is CSF flow in the dorsal SAS and the blue line is CSF flow in the ventral SAS. Solid lines are mean, and shaded region is ± one SD. Negative CSF flow is in the caudal direction (systolic flow). *CSF* cerebrospinal fluid, *PC-MRI* phase-contrast magnetic resonance imaging, *SD* standard deviation, *SAS* subarachnoid space

Bidirectional (cranial-caudal) CSF flow across the cardiac cycle was observed at all spinal levels (Fig. 2). At the thoracolumbar levels, the flow waveform had similar shape and amplitude for all animals. The standard deviation at C2/C3 across the cardiac cycle was larger than at the other spinal levels. The flow waveforms at C2/C3 start at the beginning of the diastolic pulse due to peripheral pulse gating [46]. Stroke volume at C2/C3 was 0.09 ± 0.04 mL/cycle in the dorsal SAS, and 0.04 ± 0.03 mL/cycle in the ventral SAS. At L1/L2, stroke volume was 0.02 ± 0.01 mL/cycle in the dorsal SAS, and 0.04 ± 0.04 mL/cycle in the ventral SAS. Stroke volume and net flow data for all spinal levels are available in Additional file 1: Table S5.

**Fig. 3:**
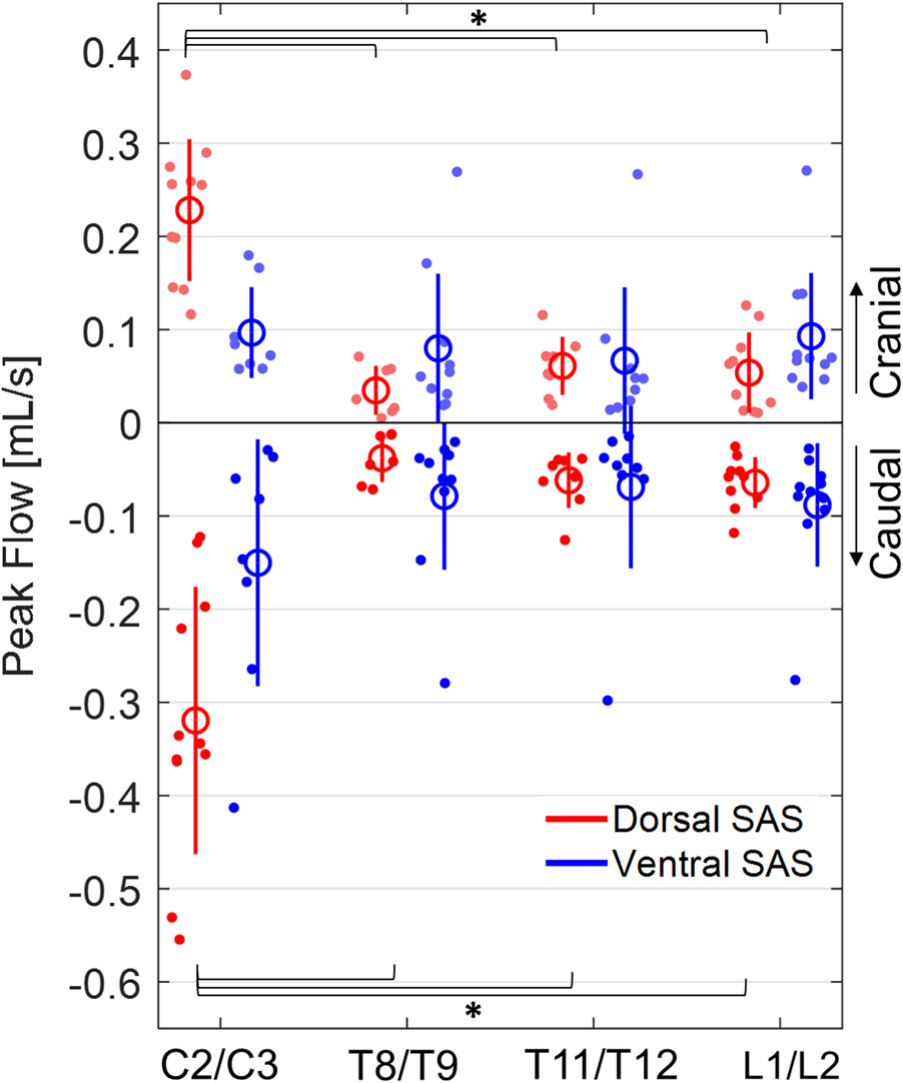
Peak CSF flow during systole (caudal) and diastole (cranial) at four spinal levels (n = 11). The red data is CSF flow in the dorsal SAS and the blue data is CSF flow in the ventral SAS. Significant differences (*) between spinal levels for the select SAS region, correspond to LMM post-hoc pairwise comparisons (p ≤ 0.05). Each data point is from one animal, large circle presents the mean ± one SD (solid line). *CSF* cerebrospinal fluid, *SAS* subarachnoid space, *LMM* linear mixed-effects models, *SD* standard deviation

The effect of spinal level on peak systolic flow was dependent on SAS region (p < 0.001) (Fig. 3). In the dorsal SAS, it was greater at C2/C3 (dorsal: -0.32 ± 0.14) compared to the thoracolumbar levels: T8/T9 (-0.04 ± 0.03 mL/s, p < 0.001), T11/T12 (-0.03 ± 0.06 mL/s, p < 0.001), L1/L2 (-0.03 ± 0.06 mL/s, p < 0.001). In the ventral SAS, there was no difference in peak systolic flow between C2/C3 (-0.15 ± 0.13 mL/s) and the thoracolumbar levels: T8/T9 (-0.08 ± 0.08 mL/s, p = 0.275), T11/T12 (-0.07 ± 0.09 mL/s, p = 0.258), and L1/L2 (-0.09 ± 0.07 mL/s, p = 0.511). In both SAS regions there were no differences in peak systolic flow between T8/T9 and T11/T12 (dorsal and ventral: p = 1.000), and T11/T12 and L1/L2 (dorsal and ventral: p = 1.000).

The effect of spinal level on peak diastolic flow was dependent on SAS region (p < 0.001). Peak diastolic flow in the dorsal SAS was greater at C2/C3 (0.29 ± 0.08 mL/s) than the thoracolumbar levels: T8/T9 (0.03 ± 0.03 mL/s, p < 0.001), T11/T12 (dorsal: 0.06 ± 0.03 mL/s, p < 0.001), L1/L2 (dorsal: 0.05 ± 0.04 mL/s, p < 0.001). In the ventral SAS, there was no difference in peak diastolic flow between C2/C3 (0.10 ± 0.05 mL/s) and the thoracolumbar levels: T8/T9 (0.08 ± 0.08 mL/s, p = 1.000), T11/T12 (0.07 ± 0.08 mL/s, p = 1.000), and L1/L2 (0.09 ± 0.07 mL/s, p = 1.000). There were no differences in both SAS regions, between T8/T9 and T11/T12 (dorsal and ventral: p = 1.000) and T11/T12 and L1/L2 (dorsal and ventral: p = 1.000). Estimated marginal means are available in Additional file 1: Table S6, and complete pairwise comparisons are available in Additional file 1: Table S7.

### Maximum CSF velocity

**Fig. 4:**
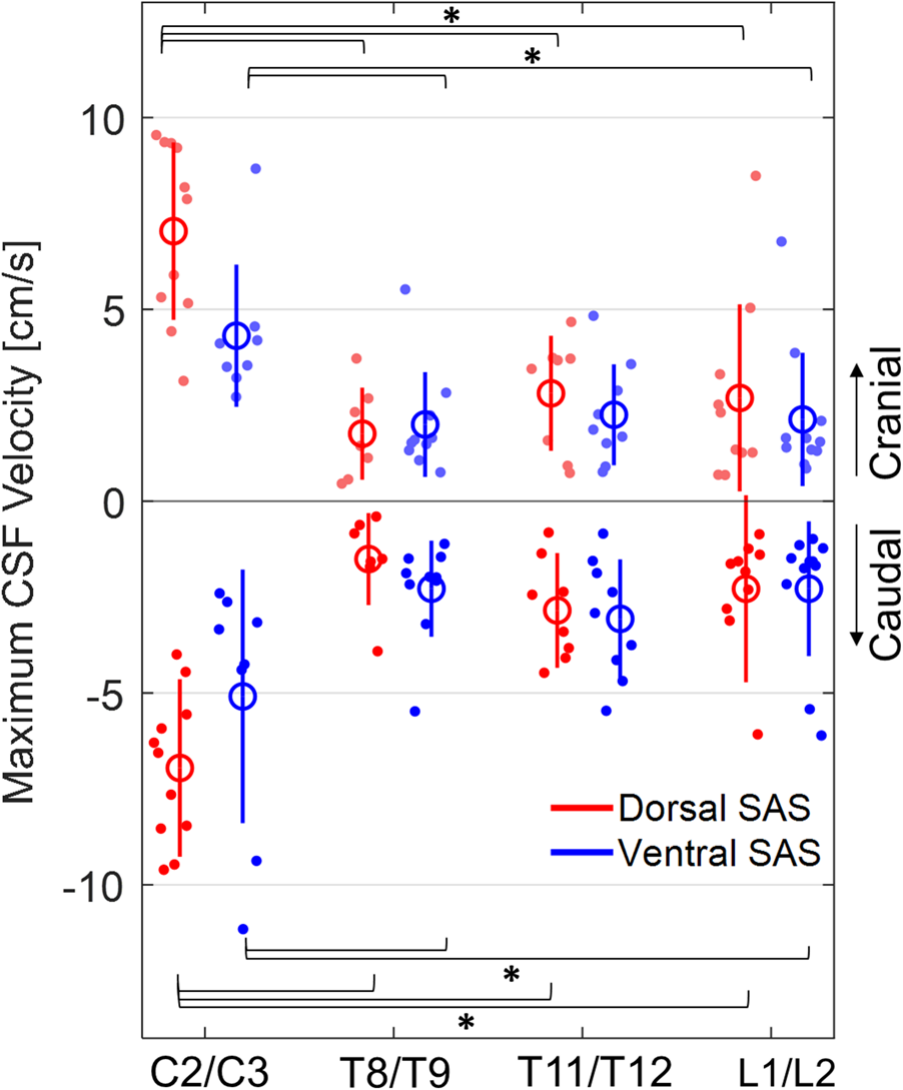
Maximum CSF velocity in the cranial and caudal direction in the SAS at four spinal levels (n = 11). The red data is CSF flow in the dorsal SAS and the blue data is CSF flow in the ventral SAS. Significant differences (*) between spinal levels for the select SAS region, correspond to LMM post-hoc pairwise comparisons (p ≤ 0.05). Each data point is from one animal, large circle presents the mean ± one SD (solid line). *CSF* cerebrospinal fluid, *SAS* subarachnoid space, *LMM* linear mixed-effects models, *SD* standard deviation

The effect of spinal level on maximum CSF velocity in the caudal direction was dependent on SAS region (p = 0.009) (Fig. 4). In the dorsal SAS, maximum CSF velocity in the caudal direction was greater at C2/C3 (− 6.95 ± 2.31 cm/s) than at each of the thoracolumbar levels: T8/T9 (− 1.51 ± 1.20 cm/s, p < 0.001), T11/T12 (− 2.85 ± 1.50 cm/s, p < 0.001), L1/L2 (− 2.28 ± 2.44 cm/s, p < 0.001). In the ventral SAS, maximum CSF velocity in the caudal direction was also greater at C2/C3 (− 5.09 ± 3.30 cm/s) than at T8/T9 (-2.28 ± 1.25 cm/s, p < 0.001), and L1/L2 (-2.28 ± 1.76 cm/s, p < 0.001), but there was no difference between C2/C3 and T11/T12 (− 3.07 ± 1.55 cm/s, p = 0.146). There were no differences in maximum CSF velocity between T8/T9 and T11/T12 (dorsal: p = 0.230, ventral: p = 0.651), and T11/T12 and L1/L2 (dorsal: p = 1.000, ventral: p = 0.641), in both SAS regions.

The effect of spinal level on maximum CSF velocity in the cranial direction was dependent on SAS region (p = 0.003). In the dorsal SAS, maximum CSF velocity in the cranial direction was greater at C2/C3 (7.04 ± 2.31 cm/s) than the thoracolumbar levels: T8/T9 (1.76 ± 1.20 cm/s, p < 0.001), T11/T12 (2.81 ± 1.50 cm/s, p < 0.001), L1/L2 (2.69 ± 2.44 cm/s, p < 0.001). In the ventral SAS, maximum CSF velocity in the cranial direction was greater at C2/C3 (4.31 ± 1.9 cm/s) than T8/T9 (2.0 ± 1.37 cm/s, p = 0.016), and L1/L2 (2.13 ± 1.74 cm/s, p = 0.022), but there was no difference between C2/C3 and T11/T12 (2.25 ± 1.32 cm/s, p = 0.178). There were no differences in maximum CSF velocity in the cranial direction between T8/T9 and T11/T12 (dorsal: p = 0.445; ventral: p = 1.000), and T11/T12 and L1/L2 (dorsal: p = 1.000; ventral: p = 1.000) in both regions. Estimated marginal means are available in Additional file 1: Table S6, and complete pairwise comparisons are available in Additional file 1: Table S7.

### Velocity wave propagation speed

**Fig. 5:**
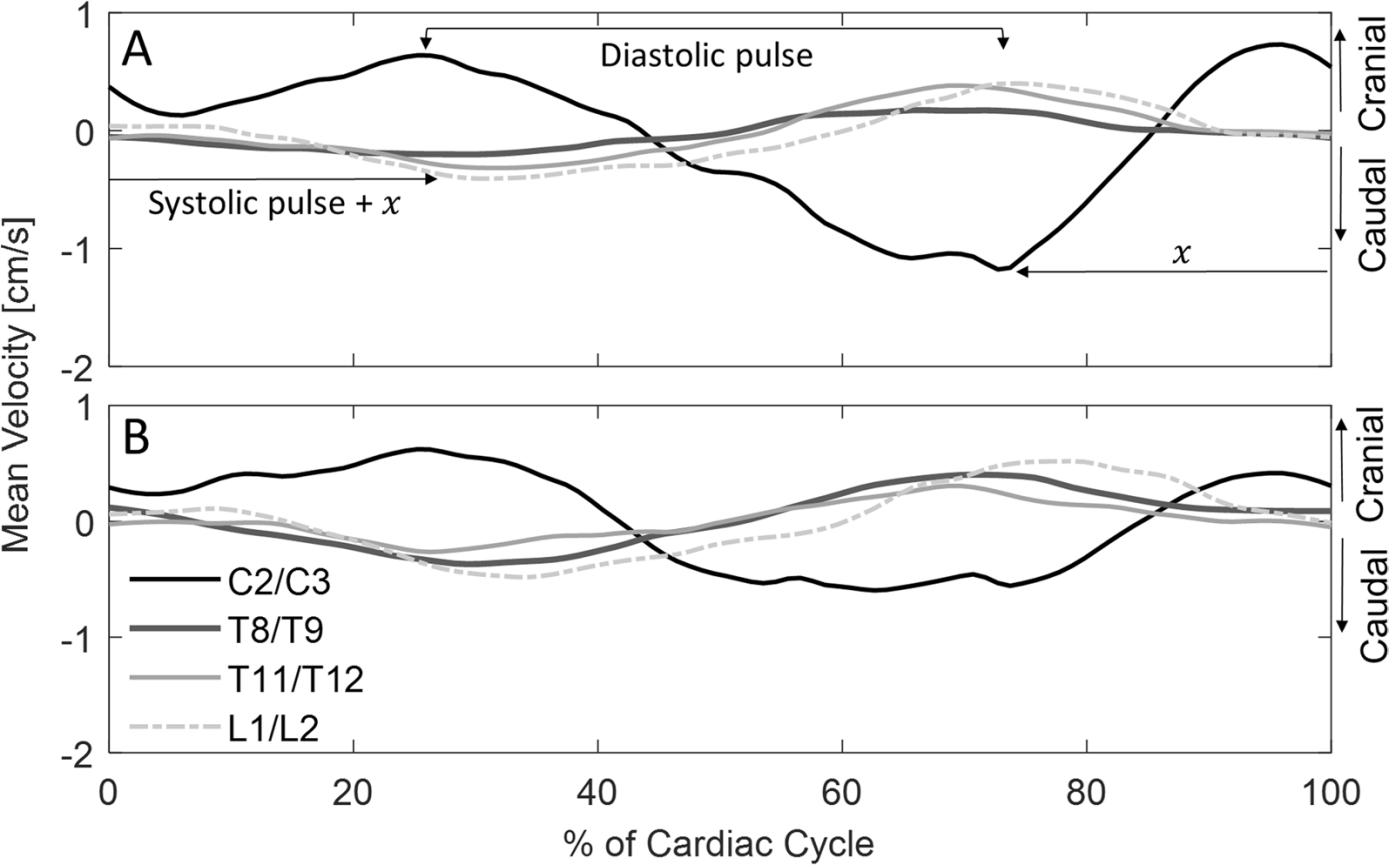
Mean velocity waveforms (n = 9) over one cardiac cycle for four spinal levels in the **A** dorsal SAS and the **B** ventral SAS. Waveforms indicate velocity wave propagation from C2/C3 to L1/L2. *SAS* subarachnoid space

The mean CSF velocity waveforms (Additional file 1: Fig. S3) demonstrated a phase shift from C2/C3 to L1/L2, indicating propagation of the velocity wave in the caudal direction in both the dorsal and ventral regions (Fig. 5A, B). The mean distance between C2/C3 and L1/L2 was 416 ± 24 mm. Peak systolic velocity at C2/C3 was close to coincident with peak diastolic CSF velocity at T8/T9, T11/T12 and L1/L2 (Additional file 1: Table S9). Peak systolic velocity at C2/C3 occurred at 70.0 ± 6.3% (392.9 ± 35.5 ms) of the cardiac cycle in the dorsal SAS and at 64.8 ± 9.3% (341.1 ± 49.1 ms) of the cardiac cycle in the ventral SAS. Peak diastolic velocity at L1/L2 occurred at 74.9 ± 10.3% (391.3 ± 54.0 ms) of the cardiac cycle in the dorsal SAS, and at 80.0 ± 7.4% (405.4 ± 37.5 ms) in the ventral SAS. Systolic (caudal) VWS (C2/C3 to L1/L2) was 1.02 ± 0.25 m/s in the dorsal SAS and 0.91 ± 0.22 m/s in the ventral SAS, and diastolic (cranial) VWS was 1.41 ± 0.39 m/s in the dorsal SAS and 1.22 ± 0.21 m/s in the ventral SAS (Fig. 5).

## Discussion

CSF dynamics along the spine have previously been characterised in humans and NHP (macaca fascicularis) [14, 15, 17, 22–26]. Large animal models are increasingly being used to investigate SCI [35, 40]. The current study provides normative data for spinal CSF flow in experimentally naïve anaesthetised domestic pigs as a baseline and comparison for future SCI studies. The study provides evidence that spinal CSF flow in anaesthetised and ventilated pigs is lower, and has slower velocity wave propagation, than in healhy, conscious humans [22, 44, 47], but is similar to that in anaesthetised NHP [26].

Spinal CSF flow in anaesthetised pigs is considerably lower than in healthy, conscious humans across the cardiac cycle. CSF flow measured in the cervical (C2–C4) spine of awake, healthy humans at peak systole is 2.4–3.5 mL/s, and at peak diastole is 1.5–2.8 mL/s [15, 19, 22, 48], which is approximately seven times greater than in the anaesthetised pigs. CSF flow in the lumbar spine (L1–L3) in healthy humans at peak systole is 1.07–1.26 mL/s, and at peak diastole is 0.29–0.58 mL/s [22, 76], which is approximately eight and four times greater, respectively, than in the pigs. In addition, stroke volume in the cervical (C2) spine in healthy humans is approximately 0.5 mL/cycle [49, 50], which is four times greater than in the pigs. In healthy conscious humans, peak mean velocity in the thoracic region (5.81 ± 1.42 cm/s; N = 14) [51] is greater than in the anaesthetised pig (Additional file 1: Table S10). The SAS is also larger in humans than the pig: for example, the T10 anterior–posterior diameter of the CSF (dural minus spinal cord diameter) in the domestic pig is 2.09 mm [1.70 mm–2.66 mm] (median and range; ultrasound measurements) [52], whereas in humans it is 7.4 ± 3.1 mm (mean ± SD; computed tomography contrast myelogram measurements) [53]. Together, these results suggest that higher CSF flow in conscious humans than anaesthetised pigs results, at least in part, from the combined effect of a larger SAS and greater CSF velocity in humans. CSF flow in the pig was similar to that reported for anaesthetised, but not mechanically ventilated, NHPs: NHP peak systolic flow in the cervical spine (0.5 ± 0.2 mL/s, C2/C3; estimated from graphical data), and in the lumbar spine (0.1 ± 0.05 mL/s, L3/L4; estimated from graphical data), was approximately the same as in the pigs. NHP cervical peak diastolic flow was nearly two times smaller than (0.2 ± 0.2 mL/s; estimated from graphical data), and lumbar flow was approximately the same as (0.1 ± 0.1 mL/s; estimated from graphical data), that in the current pig study [26]. In pigs, peak systolic CSF flow decreased from the cervical to the thoracic levels, but was unchanged at the thoracolumbar levels both dorsally and ventrally. In humans, peak systolic and diastolic flow decreases caudally to a minimum in the lumbar region [22]. Such a reduction across the spine may not have been observed in the current study because the thoracic (T8/T9) and lumbar (L1/L2) measurements encompassed a limited span (193 ± 20 mm). It is also possible that respiratory forces have a greater effect on CSF flow in the thoracic region compared to at the lumbar levels [23], and that no difference was observed between adjacent thoracic and lumbar sites due to mechanical ventilation of the pigs.

Although the relative contribution of respiratory and cardiac cycles to CSF dynamics is unclear, it is evident that physiological variations of both can alter CSF flow [54–57]. It is commonly thought that CSF travels cranially with inhalation, and caudally with exhalation, due to changes in intrathoracic pressure with spontaneous breathing [23, 24, 55, 56]. Unlike in most non-acute clinical CSF flow studies reported, the animals in this study were anesthetised and ventilated, which causes positive intrathoracic pressure and produces increased pressure during inhalation and decreased pressure during exhalation (the opposite of spontaneous breathing). Positive intrathoracic pressure throughout the respiratory cycle may contribute to lower CSF flow. Mechanically ventilated rats had less movement of fluorescent tracer in the spinal SAS in the caudal direction compared to spontaneously breathing animals [54]. This suggests that respiratory conditions need to be carefully considered in CSF flow study comparisons. Mechanical ventilation can also influence arterial pulse pressure through complex cardiopulmonary interactions [58]. It is apparent that reduced arterial pulse pressure decreases CSF flow in the spinal perivascular spaces [59, 60], but its effect on CSF pulsations in the SAS is unknown. There is limited and conflicting evidence that heart rate influences CSF flow: increasing heart rate resulted in increased bidirectional CSF flow velocities in a three dimensional computational fluid dynamics model of the SAS [57], while a study in rats showed that increased heart rate had little influence on CSF flow [54]. The heart rate range in this study (71–174 bpm) is larger than in the other animal studies (canines, 70–110 bpm [73] ; NHP, 92–132 bpm [26]). In this current study, two animals had notably higher heart rates (P004, 120–130 bpm; P012, 174 bpm), both with apparent abnormal cardiac gating. The animal (P006) with markedly higher peak systolic and diastolic CSF flow across most spinal levels in the ventral region (Additional file 1: Table S3) had unremarkable physiological parameters (Additional file 1: Table S4). Anaesthesia may also contribute to CSF dynamics [61], and this is potentially due to its influence on blood pressure and partial pressure of carbon dioxide in the blood stream. Mean arterial pressure is thought to influence CSF flow in the cerebral perivascular spaces [62]; however, its effect on pulsating spinal CSF flow in the SAS is understudied. In one rat study there was no effect of mean arterial pressure on spinal SAS CSF flow [54]. In the current study, the animal that received a propofol bolus during the scan (P014; T11/T12 and L1/L2 scanned prior to bolus, and T8/T9 and C2/C3 after bolus) had abnormally higher peak systolic flow at T11/T12 and L1/L2 dorsally, T8/T9 and L1/L2 ventrally and peak diastolic flow at T8/T9 dorsally. The propofol bolus was indicated because the animal was not deeply anaesthetised (as evidenced by physical movement), and was therefore likely to have higher blood pressure; however, blood pressure was not measured concurrently in this study. In addition, cerebral blood flow is influenced by the partial pressure of arterial carbon dioxide [63]. Hypercapnia increases cerebral blood flow and intracranial pressure [64], which may effect spinal CSF flow due to the potential interaction between intracerebral arteries and pulse propagation [65]. It is becoming increasingly clear that respiratory forces have a large influence on CSF flow; however, the potential contributions of other physiological parameters such as heart rate, blood pressure, and carbon dioxide in the bloodstream, need to be further investigated to establish their relative effect on CSF dynamics. These physiological parameters should be measured and recorded during PC-MRI acquisition of CSF flow in future studies.

Velocity wave propagation was detected in the caudal direction along the spinal axis in laterally recumbent pigs. Velocity wave propagation speed can be used to approximate pulse wave velocity (pressure wave propagation) since it has been shown that they are nearly identical under certain conditions [47]. Because of this, some studies use the term ‘pulse wave velocity’ rather than VWS, for the identical measure [26, 44]. It has been hypothesised that CSF pressure waves originate in the intracerebral arteries and propagate in a caudal direction [65]; however, the origin of the pulse remains unresolved since there are other studies which suggest local sources [22, 66, 67]. In cardiovascular diagnostics, pulse wave velocity is a measure of vessel compliance. VWS has recently been reported for spinal CSF flow studies [44, 47, 68] where it likely reflects compliance of the spinal cord tissue, dura, and surrounding tissues [69]. A one-dimensional tube model of the spinal SAS has been used to show that increasing spinal compliance results in slower VWS and greater attenuation of the pulse [70]. As observed in these data (Fig. 5), spinal compliance can enable CSF to flow in opposite directions concurrently at spinal locations remote to each other (e.g. C2/C3 and L1/L2). In healthy conscious humans, VWS is three (4.6 ± 1.7 m/s)[47] to four (5.83 ± 3.36 m/s) [44] times faster than that measured in the pigs. In NHP, VWS is similar to the VWS estimated for the dorsal SAS in this study (1.13 m/s) [26]. In humans, compliance provided by the craniospinal compartment compensates for postural pressure changes [49, 50]. The extent of this effect in quadrupeds is unclear. In the current study, VWS was calculated between C2/C3 and L1/L2, rather than between each adjacent spinal levels. Because the lower spinal levels sampled were concentrated around T10, the increased distance, and therefore pulse transit times, between these locations should improve temporal accuracy but remove detection of region-specific VWS. Further study is necessary to elucidate the relevance of VWS along the spine, and locally, in healthy and diseased states.

Defining dorsal and ventral regions in the SAS is likely to be beneficial for future CSF flow investigations in the context of SCI, where SAS occlusion may not be uniformly distributed. The current study suggests that, in these pigs, CSF flow and maximum velocity were dependent on SAS region at C2/C3. While the majority of studies do not report CSF flow in separate SAS regions, a study using dynamic sagittal PC-MRI scans on cervical myelopathy patients found that grade 2 cervical stenosis was more frequently associated with interrupted flow patterns in the ventral or dorsal SAS [27]. A study of Chiari malformation in canines reported that syringomyelia was associated with lower peak velocity in the dorsal SAS (foramen magnum and C2/C3) but not in the ventral SAS [71]. Together, these observations suggest the potential importance of considering local CSF flow characteristics. In the current study, CSF flow was generally similar, but not identical, in the two regions, and at spinal level T8/T9, four animals had no flow signal in the dorsal SAS while CSF flow was observed in the ventral SAS. There is some evidence that CSF velocity is higher ventrally during both systolic and diastolic flow, in the human cervical SAS [72]; therefore, it is possible that in these animals flow in the dorsal SAS was not detected by PC-MRI because of lower velocity. In addition, the variability of maximum CSF velocity between animals in this study suggests that multi-V_ENC_ scans should be run in the thoracolumbar spine. Maximum CSF velocity at C2/C3 also exceeded the applied V_ENC_ (6 cm/s) in 70% of the scans in the first ten animals. Aliasing can be difficult to detect prior to image post-processing, therefore “real-time” post-processing should be completed immediately after the scan to allow for immediate re-scanning as needed. A V_ENC_ of at least 8 cm/s at C2/C3 is recommended to minimise the risk of aliasing, in pigs.

There are several limitations in this study. The sample size was limited; however, the number of animals used in this study is greater than that reported for similar animal studies characterising CSF flow in NHP (macaca fascicularis; N = 8) [26], and quantifying CSF velocity in canines (beagle, N = 6) [73]. In addition, the age and sex of the animals in this study were selected because of their relevance to the associated SCI experiments; it is not known if CSF flow characteristics change with animal age, size and sex. The spinal levels selected were of particular relevance to pig SCI contusion models, and may not be generalisable to levels of interest for the study of other spinal pathologies. Blood pressure was not measured concurrently with image acquisition, so blood pressure could not be identified as a physiological contributor to variability in CSF flow. Because the SAS was split into two regions with different flow, eddy current corrections were optimised for the total net flow of both regions, rather than for each region. When performing eddy current correction, we assumed that the net flow over a single cardiac cycle was zero. Although it is understood that the majority of CSF is formed cranially and net flow must occur since it is absorbed at the same rate, this assumption is likely acceptable because net flow is very low across a single cardiac cycle. Estimated CSF formation is approximately 0.067 mL/min in the pig (based on data from 25–30 kg female sheep [74]), and therefore, the volume of CSF produced (and absorbed) during one cardiac cycle (at 100 bpm) is approximately 0.67 µL. The cause of the apparent abnormal cardiac gating in two animals could not be identified; the pulse oximeter signal was not acquired for post-processing. Although the cardiac phase numbers were adjusted for heart rate to optimise competing demands of temporal resolution and signal-to-noise ratio, the minimum number of phases selected in this study was relatively low and could be increased in future studies. The temporal resolution of the acquired cardiac phases varied from 16 to 41 ms, which may have affected the temporal outcome parameters [75]. In addition, the cause of undetected CSF flow in three animals could not be identified; however, technical failures and/or insufficient CSF flow have also been reported in human PC-MRI studies [22, 24, 44, 68].

## Conclusions

This study demonstrates that CSF flow in the pig can be detected and quantified with PC-MRI using a 3T MR scanner. Spinal CSF flow in the healthy, anaesthetised, ventilated domestic pig in lateral recumbency is lower, and has a lower velocity wave propagation speed, than in conscious humans. These data are a normative baseline at spinal levels relevant for future SCI research in this animal model, and provide a means to validate computational models of the pig SAS.

## Supplementary Information

**Additional file 1: Figure S1.** Exemplar PC-MRI **A** magnitude **B** phase images at peak diastolic flow with two ROIs in the dorsal SAS dependent on flow signal within that region (P009). **Table S1. **Net flow (mL/cycle) and percent residual flow (%) for each animal and spinal level, with means ± one standard deviation. **Table S2.** The number of ROI and area of ROI (mm^2^) drawn in the dorsal or ventral SAS dependent on detectable CSF flow signal within that region, where 0 corresponds to no CSF flow detected in that region. **Figure S2.** Experimental flow data from P004 and P012 were excluded from temporal analyses and plots due to abnormal cardiac gating. **Table S3.** Peak systolic and diastolic flow values (mL/s) for each animal and spinal level, with means ± one standard deviation. **Table S4.** Physiological recordings and anaesthetic drug levels of each animal during the PC-MRI scan. **Table S5.** Stroke volumes (mL/cycle) for each animal and spinal level, with means ± one standard deviation. **Table S6.** Estimated marginal means with 95% CI for peak diastolic and systolic flow, and maximum cranial and caudal velocity from LMMs. **Table S7.** Summary of results from the LMM pairwise comparisons for peak systolic and diastolic flow and maximum cranial and caudal velocity. **Figure S3.** Mean CSF velocity in the dorsal and ventral region of the SAS at each spinal level. **Table S8. **Time to peak systolic and diastolic velocity (as a percentage of the cardiac cycle) and cardiac cycle duration (ms) for each animal and spinal level. **Table S9.** Peak mean systolic and diastolic velocity values (mL/s) for each animal and spinal level, with means ± one standard deviation.

## Data Availability

The datasets generated and/or analysed during the current study are available in the Figshare repository, 0.25909/20494356.

## Abbreviations

CI: Confidence interval
CNS: Central nervous system
CSF: Cerebrospinal fluid
C2/C3: Cervical spine between levels 2 and 3
LMM: Linear mixed-effects model
L1/L2: Lumbar spine between levels 1 and 2
MRI: Magnetic resonance imaging
NHP: Non-human primates
PC-MRI: Phase-contrast magnetic resonance imaging
ROI: Region of interest
SAS: Subarachnoid space
SCI: Spinal cord injury
SD: Standard deviation
T8/T9: Thoracic spine between levels 8 and 9
T11/T12: Thoracic spine between levels 11 and 12
VWS: Velocity wave speed

## References

[1] Proulx ST (2021). Cerebrospinal fluid outflow: a review of the historical and contemporary evidence for arachnoid villi, perineural routes, and dural lymphatics. CMLS.

[2] Lindstrøm EK, Ringstad G, Mardal K-A, Eide PK (2018). Cerebrospinal fluid volumetric net flow rate and direction in idiopathic normal pressure hydrocephalus. Neuroimage Clin.

[3] Ringstad G, Emblem KE, Eide PK (2016). Phase-contrast magnetic resonance imaging reveals net retrograde aqueductal flow in idiopathic normal pressure hydrocephalus. JNS.

[4] Luetmer PH, Huston J, Friedman JA, Dixon GR, Petersen RC, Jack CR (2002). Measurement of cerebrospinal fluid flow at the cerebral aqueduct by use of phase-contrast magnetic resonance imaging: technique validation and utility in diagnosing idiopathic normal pressure hydrocephalus. Neurosurgery.

[5] Haughton VM, Korosec FR, Medow JE, Dolar MT, Iskandar BJ (2003). Peak systolic and diastolic CSF velocity in the foramen magnum in adult patients with Chiari I malformations and in normal control participants. AJNR Am J Neuroradiol.

[6] Bunck AC, Kroeger JR, Juettner A, Brentrup A, Fiedler B, Crelier GR (2012). Magnetic resonance 4D flow analysis of cerebrospinal fluid dynamics in Chiari I malformation with and without syringomyelia. Eur Radiol.

[7] Khani M, Burla GKR, Sass LR, Arters ON, Xing T, Wu H (2022). Human in silico trials for parametric computational fluid dynamics investigation of cerebrospinal fluid drug delivery: impact of injection location, injection protocol, and physiology. Fluids and Barriers of the CNS.

[8] Naseri Kouzehgarani G, Feldsien T, Engelhard HH, Mirakhur KK, Phipps C, Nimmrich V (2021). Harnessing cerebrospinal fluid circulation for drug delivery to brain tissues. Adv Drug Deliv Rev.

[9] Takizawa K, Matsumae M, Hayashi N, Hirayama A, Yatsushiro S, Kuroda K (2017). Hyperdynamic CSF motion profiles found in idiopathic normal pressure hydrocephalus and Alzheimer’s disease assessed by fluid mechanics derived from magnetic resonance images. Fluids and Barriers of the CNS.

[10] Berliner JA, Woodcock T, Najafi E, Hemley SJ, Lam M, Cheng S (2019). Effect of extradural constriction on CSF flow in rat spinal cord. Fluids Barriers CNS.

[11] Yeo J, Cheng S, Hemley S, Lee BB, Stoodley M, Bilston L (2017). Characteristics of CSF Velocity-Time Profile in Posttraumatic Syringomyelia. Am J Neuroradiol.

[12] Spijkerman JM, Geurts LJ, Siero JCW, Hendrikse J, Luijten PR, Zwanenburg JJM (2019). Phase contrast MRI measurements of net cerebrospinal fluid flow through the cerebral aqueduct are confounded by respiration. J Magn Reson Imaging.

[13] Wymer DT, Patel KP, Bhatia VK (2020). Phase-contrast MRI: physics, techniques, and clinical applications. Radiographics.

[14] Enzmann D, Pelc N (1991). Normal flow patterns of intracranial and spinal cerebrospinal fluid defined with phase-contrast cine MR imaging. Radiology.

[15] Henry-Feugeas MC, Idy-Peretti I, Blanchet B, Hassine D, Zannoli G, Schouman-Claeys E (1993). Temporal and spatial assessment of normal cerebrospinal fluid dynamics with MR imaging. Magn Reson Imaging.

[16] Greitz D (1993). Cerebrospinal fluid circulation and associated intracranial dynamics. A radiologic investigation using MR imaging and radionuclide cisternography. Acta Radiol Suppl.

[17] Alperin N, Vikingstad EM, Gomez-Anson B, Levin DN (1996). Hemodynamically independent analysis of cerebrospinal fluid and brain motion observed with dynamic phase contrast MRI. Magn Reson Med.

[18] Bradley WG, Scalzo D, Queralt J, Nitz WN, Atkinson DJ, Wong P (1996). Normal-pressure hydrocephalus: evaluation with cerebrospinal fluid flow measurements at MR imaging. Radiology.

[19] Balédent O, Gondry-Jouet C, Meyer M-E, De Marco G, Le Gars D, Henry-Feugeas M-C (2004). Relationship between cerebrospinal fluid and blood dynamics in healthy volunteers and patients with communicating hydrocephalus. Invest Radiol.

[20] Dolar MT, Haughton VM, Iskandar BJ, Quigley M (2004). Effect of craniocervical decompression on peak CSF velocities in symptomatic patients with Chiari I malformation. AJNR Am J Neuroradiol.

[21] Clarke EC, Stoodley MA, Bilston LE (2013). Changes in temporal flow characteristics of CSF in Chiari malformation Type I with and without syringomyelia: implications for theory of syrinx development: Clinical article. JNS.

[22] Bert RJ, Settipalle N, Tiwana E, Muddasani D, Nath R, Wellman B (2019). The relationships among spinal CSF flows, spinal cord geometry, and vascular correlations: evidence of intrathecal sources and sinks. Am J Physiol Regul Integr Comp Physiol.

[23] Aktas G, Kollmeier JM, Joseph AA, Merboldt K-D, Ludwig H-C, Gärtner J (2019). Spinal CSF flow in response to forced thoracic and abdominal respiration. Fluids and barriers of the CNS..

[24] Dreha-Kulaczewski S, Konopka M, Joseph AA, Kollmeier J, Merboldt K-D, Ludwig H-C (2018). Respiration and the watershed of spinal CSF flow in humans. Sci Rep.

[25] Yan L, Liu H, Shang H (2012). Quantitative analysis of intraspinal cerebrospinal fluid flow in normal adults. Neural Regen Res.

[26] Khani M, Lawrence BJ, Sass LR, Gibbs CP, Pluid JJ, Oshinski JN (2019). Characterization of intrathecal cerebrospinal fluid geometry and dynamics in cynomolgus monkeys (macaca fascicularis) by magnetic resonance imaging. PLoS ONE.

[27] Bae YJ, Lee JW, Lee E, Yeom JS, Kim K-J, Kang HS (2017). Cervical compressive myelopathy: flow analysis of cerebrospinal fluid using phase-contrast magnetic resonance imaging. Eur Spine J.

[28] Shibuya R, Yonenobu K, Koizumi T, Kato Y, Mitta M, Yoshikawa H (2002). Pulsatile cerebrospinal fluid flow measurement using phase-contrast magnetic resonance imaging in patients with cervical myelopathy. Spine.

[29] Tominaga T, Watabe N, Takahashi T, Shimizu H, Yoshimoto T (2002). Quantitative assessment of surgical decompression of the cervical spine with cine phase contrast magnetic resonance imaging. Neurosurgery.

[30] Kim H-J, Kim H, Kim Y-T, Sohn C-H, Kim K, Kim D-J (2021). Cerebrospinal fluid dynamics correlate with neurogenic claudication in lumbar spinal stenosis. PLoS ONE.

[31] Saadoun S, Werndle MC, Lopez de Heredia L, Papadopoulos MC. The dura causes spinal cord compression after spinal cord injury. Br J Neurosurg. 2016;30(5):582–4. 10.3109/02688697.2016.1173191 27080553

[32] Leypold BG, Flanders AE, Burns AS (2008). The early evolution of spinal cord lesions on MR imaging following traumatic spinal cord injury. AJNR Am J Neuroradiol.

[33] Kim SY, Shin MJ, Chang JH, Lee CH, Shin YI, Shin YB (2015). Correlation of diffusion tensor imaging and phase-contrast MR with clinical parameters of cervical spinal cord injuries. Spinal Cord.

[34] Yeo J, Cheng S, Hemley S, Lee BB, Stoodley M, Bilston L (2017). Characteristics of CSF Velocity-Time Profile in Posttraumatic Syringomyelia. AJNR Am J Neuroradiol.

[35] Kwon BK, Streijger F, Hill CE, Anderson AJ, Bacon M, Beattie MS (2015). Large animal and primate models of spinal cord injury for the testing of novel therapies. Exp Neurol.

[36] Busscher I, Ploegmakers JJ, Verkerke GJ, Veldhuizen AG (2010). Comparative anatomical dimensions of the complete human and porcine spine. Eur Spine J.

[37] Jones CF, Cripton PA, Kwon BK (2012). Gross morphological changes of the spinal cord immediately after surgical decompression in a large animal model of traumatic spinal cord injury. Spine (Phila Pa 1976).

[38] Toossi A, Bergin B, Marefatallah M, Parhizi B, Tyreman N, Everaert DG (2021). Comparative neuroanatomy of the lumbosacral spinal cord of the rat, cat, pig, monkey, and human. Sci Rep.

[39] Sun Y, Zhang LH, Fu YM, Li ZR, Liu JH, Peng J (2016). Establishment of a rat model of chronic thoracolumbar cord compression with a flat plastic screw. Neural Regen Res.

[40] Lee JH, Jones CF, Okon EB, Anderson L, Tigchelaar S, Kooner P (2013). A novel porcine model of traumatic thoracic spinal cord injury. J Neurotrauma.

[41] National Health and Medical Research Council (2013). Australian code for the care and use of animals for scientific purposes.

[42] Heiberg E, Sjögren J, Ugander M, Carlsson M, Engblom H, Arheden H (2010). Design and validation of Segment–freely available software for cardiovascular image analysis. BMC Med Imaging.

[43] Battal B, Kocaoglu M, Bulakbasi N, Husmen G, Tuba Sanal H, Tayfun C (2011). Cerebrospinal fluid flow imaging by using phase-contrast MR technique. Br J Radiol.

[44] Sonnabend K, Brinker G, Maintz D, Bunck AC, Weiss K (2021). Cerebrospinal fluid pulse wave velocity measurements: In vitro and in vivo evaluation of a novel multiband cine phase-contrast MRI sequence. Magn Reson Med.

[45] Koo TK, Li MY (2016). A guideline of selecting and reporting intraclass correlation coefficients for reliability research. J Chiropr Med.

[46] Bert RJ, Settipalle N, Muddasani D, Tiwana E, Wellman B, Negahdar MJ (2020). ECG gating is more precise than peripheral pulse gating when quantifying spinal CSF pulsations using phase contrast cine MRI. Acad Radiol.

[47] Kalata W, Martin BA, Oshinski JN, Jerosch-Herold M, Royston TJ, Loth F (2009). MR measurement of cerebrospinal fluid velocity wave speed in the spinal canal. IEEE Trans Biomed Eng.

[48] Alperin N, Lee SH, Sivaramakrishnan A, Hushek SG (2005). Quantifying the effect of posture on intracranial physiology in humans by MRI flow studies. J Magn Reson Imaging.

[49] Alperin N, Burman R, Lee SH (2021). Role of the spinal canal compliance in regulating posture-related cerebrospinal fluid hydrodynamics in humans. J Magn Reson Imaging.

[50] Muccio M, Chu D, Minkoff L, Kulkarni N, Damadian B, Damadian RV (2021). Upright versus supine MRI: effects of body position on craniocervical CSF flow. Fluids Barriers CNS.

[51] Algin O, Koc U, Yalcin N (2022). Cerebrospinal fluid velocity changes of idiopathic scoliosis: a preliminary study on 3-T PC-MRI and 3D-SPACE-VFAM data. Childs Nerv Syst.

[52] Gayen C, Bessen MA, Dorrian R, Quarrington RD, Mulaibrahimovic A, O'Hare Doig R (2022). A survival model of thoracic contusion spinal cord injury in the domestic pig. J Neurotrauma.

[53] Gellad F, Rao KC, Joseph PM, Vigorito RD (1983). Morphology and dimensions of the thoracic cord by computer-assisted metrizamide myelography. AJNR Am J Neuroradiol.

[54] Liu S, Bilston LE, Flores Rodriguez N, Wright C, McMullan S, Lloyd R (2022). Changes in intrathoracic pressure, not arterial pulsations, exert the greatest effect on tracer influx in the spinal cord. Fluids Barriers CNS.

[55] Yamada S, Miyazaki M, Yamashita Y, Ouyang C, Yui M, Nakahashi M (2013). Influence of respiration on cerebrospinal fluid movement using magnetic resonance spin labeling. Fluids Barriers CNS..

[56] Dreha-Kulaczewski S, Joseph AA, Merboldt K-D, Ludwig H-C, Gärtner J, Frahm J (2017). Identification of the upward movement of human CSF in vivo and its relation to the brain venous system. J Neurosci.

[57] Linge SO, Mardal KA, Haughton V, Helgeland A (2013). Simulating CSF flow dynamics in the normal and the chiari i subarachnoid space during rest and exertion. Am J Neuroradiol.

[58] Michard F (2005). Changes in arterial pressure during mechanical ventilation. Anesthesiology.

[59] Lloyd RA, Stoodley MA, Fletcher DF, Bilston LE (2019). The effects of variation in the arterial pulse waveform on perivascular flow. J Biomech.

[60] Stoodley MA, Brown SA, Brown CJ, Jones NR (1997). Arterial pulsation-dependent perivascular cerebrospinal fluid flow into the central canal in the sheep spinal cord. J Neurosurg.

[61] Li J, Pei M, Bo B, Zhao X, Cang J, Fang F (2022). Whole-brain mapping of mouse CSF flow via HEAP-METRIC phase-contrast MRI. Magn Reson Med.

[62] Mestre H, Tithof J, Du T, Song W, Peng W, Sweeney AM (2018). Flow of cerebrospinal fluid is driven by arterial pulsations and is reduced in hypertension. Nat Commun.

[63] Sato K, Sadamoto T, Hirasawa A, Oue A, Subudhi AW, Miyazawa T (2012). Differential blood flow responses to CO_2_ in human internal and external carotid and vertebral arteries. J Physiol.

[64] van Hulst RA, Hasan D, Lachmann B (2002). Intracranial pressure, brain PCO2, PO2, and pH during hypo- and hyperventilation at constant mean airway pressure in pigs. Intensive Care Med.

[65] Adolph R, Fukusumi H, Fowler N (1967). Origin of cerebrospinal fluid pulsations. Am J Physiol Legacy Cont.

[66] Nakamura K, Urayama K, Hoshino Y (1997). Lumbar cerebrospinal fluid pulse wave rising from pulsations of both the spinal cord and the brain in humans. Spinal Cord.

[67] Henry-Feugeas M-C, Idy-Peretti I, Baledent O, Poncelet-Didon A, Zannoli G, Bittoun J (2000). Origin of subarachnoid cerebrospinal fluid pulsations: a phase-contrast MR analysis. Magn Reson Imaging.

[68] Sass LR, Khani M, Romm J, Schmid Daners M, McCain K, Freeman T (2020). Non-invasive MRI quantification of cerebrospinal fluid dynamics in amyotrophic lateral sclerosis patients. Fluids Barriers CNS.

[69] Bertram CD, Brodbelt AR, Stoodley MA (2005). The origins of syringomyelia: numerical models of fluid/structure interactions in the spinal cord. J Biomech Eng.

[70] Martin BA, Reymond P, Novy J, Balédent O, Stergiopulos N (2012). A coupled hydrodynamic model of the cardiovascular and cerebrospinal fluid system. Am J Physiol Heart Circ Physiol.

[71] Cerda-Gonzalez S, Olby NJ, Broadstone R, McCullough S, Osborne JA (2009). Characteristics of cerebrospinal fluid flow in cavalier king charles spaniels analysed using phase-contrast velocity cine magnetic resonance imaging. Vet Radiol Ultrasound.

[72] Shah S, Haughton V, del Río AM (2011). CSF flow through the upper cervical spinal canal in chiari i malformation. Am J Neuroradiol.

[73] Christen MA, Schweizer-Gorgas D, Richter H, Joerger FB, Dennler M (2021). Quantification of cerebrospinal fluid flow in dogs by cardiac-gated phase-contrast magnetic resonance imaging. J Vet Intern Med.

[74] Boulton M, Flessner M, Armstrong D, Hay J, Johnston M (1998). Determination of volumetric cerebrospinal fluid absorption into extracranial lymphatics in sheep. Am J Physiol Regul Integr Comp Physiol.

[75] Dorniak K, Heiberg E, Hellmann M, Rawicz-Zegrzda D, Wesierska M, Galaska R (2016). Required temporal resolution for accurate thoracic aortic pulse wave velocity measurements by phase-contrast magnetic resonance imaging and comparison with clinical standard applanation tonometry. BMC Cardiovasc Disord.

[76] Chun Se-Woong, Lee Hack-Jin, Nam Koong-Ho, Sohn Chul-Ho, Kim Kwang Dong, Jeong Eun-Jin, Chung Sun G., Kim Keewon, Kim Dong-Joo (2017). Cerebrospinal fluid dynamics at the lumbosacral level in patients with spinal stenosis: A pilot study. Journal of Orthopaedic Research.

