# Supplementary material for: Characterising spinal cerebrospinal fluid flow in the pig with phase-contrast magnetic resonance imaging
Source: Fluids Barriers CNS. 2023 Jan 18;20:5. doi: 10.1186/s12987-022-00401-4 (PMC9850564; doi:10.1186/s12987-022-00401-4)
Supplement: Supplementary file 1 — Additional file 1: Figure S1. Exemplar PC-MRI A magnitude B phase images at peak diastolic flow with two ROIs in the dorsal SAS dependent on flow signal within that region (P009). Table S1. Net flow (mL/cycle) and percent residual flow (%) for each animal and spinal level, with means ± one standard deviation. Table S2. The number of ROI and area of ROI (mm2) drawn in the dorsal or ventral SAS dependent on detectable CSF flow signal within that region, where 0 corresponds to no CSF flow detected in that region. Figure S2. Experimental flow data from P004 and P012 were excluded from temporal analyses and plots due to abnormal cardiac gating. Table S3. Peak systolic and diastolic flow values (mL/s) for each animal and spinal level, with means ± one standard deviation. Table S4. Physiological recordings and anaesthetic drug levels of each animal during the PC-MRI scan. Table S5. Stroke volumes (mL/cycle) for each animal and spinal level, with means ± one standard deviation. Table S6. Estimated marginal means with 95% CI for peak diastolic and systolic flow, and maximum cranial and caudal velocity from LMMs. Table S7. Summary of results from the LMM pairwise comparisons for peak systolic and diastolic flow and maximum cranial and caudal velocity. Figure S3. Mean CSF velocity in the dorsal and ventral region of the SAS at each spinal level. Table S8. Time to peak systolic and diastolic velocity (as a percentage of the cardiac cycle) and cardiac cycle duration (ms) for each animal and spinal level. Table S9. Peak mean systolic and diastolic velocity values (mL/s) for each animal and spinal level, with means ± one standard deviation. [file 12987_2022_401_MOESM1_ESM.docx]

**Additional file 1**

Additional file 1: Figure S1

Exemplar PC-MRI (A) magnitude (B) phase images at peak diastolic flow with two ROIs in the dorsal SAS dependent on flow signal within that region (P009). This was to ensure that the included pixels within the ROI reflect a CSF flow signal, and to avoid regions with no flow signal. PC-MRI = phase contrast magnetic resonance imaging


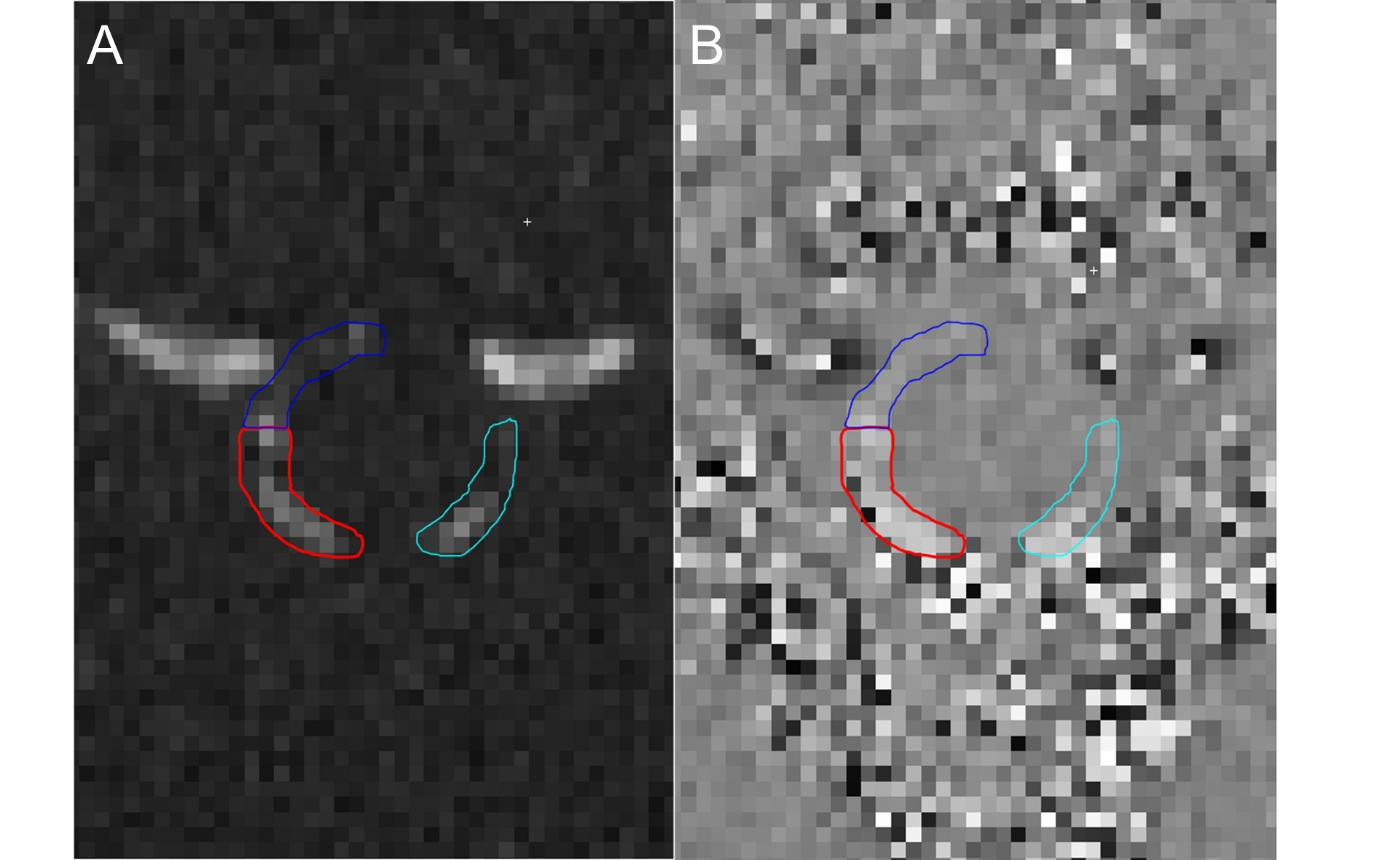


Additional file 1: Table S1

Net flow (mL/cycle) and percent residual flow (%) for each animal and spinal level, with means ± one standard deviation. SAS = subarachnoid space.

| Net flow Dorsal + Ventral SAS (mL/cycle) | | | | |
| --- | --- | --- | --- | --- |
|  | **C2/C3** | **T8/T9** | **T11/T12** | **L1/L2** |
| P001 | 0.00 | 0.00 | 0.00 | 0.00 |
| P002 | 0.00 | 0.00 | 0.00 | 0.00 |
| P003 | 0.00 | NA | NA | 0.00 |
| P006 | 0.00 | 0.00 | 0.00 | 0.00 |
| P007 | 0.00 | 0.00 | 0.00 | 0.00 |
| P008 | 0.00 | 0.00 | 0.00 | 0.00 |
| P009 | 0.00 | 0.00 | 0.00 | 0.00 |
| P010 | 0.00 | 0.00 | 0.00 | 0.00 |
| P014 | 0.00 | 0.00 | 0.00 | 0.00 |
| Mean | 0.00 | 0.00 | 0.00 | 0.00 |
| SD | 0.00 | 0.00 | 0.00 | 0.00 |
| Residual Flow Dorsal + Ventral SAS (% of total stroke volume) | | | | |
| P001 | 5.13 | 3.49 | 3.80 | 5.59 |
| P002 | 1.77 | NA | 1.65 | 0.50 |
| P003 | 2.40 | NA | NA | 7.11 |
| P006 | 1.20 | 0.29 | 1.06 | 0.44 |
| P007 | 1.49 | 1.48 | 7.50 | 3.88 |
| P008 | 3.41 | 2.31 | 0.07 | 3.60 |
| P009 | 0.64 | 8.97 | 0.00 | 4.79 |
| P010 | 7.62 | 2.18 | 0.29 | 5.32 |
| P014 | 0.43 | 0.64 | 3.25 | 2.58 |
| Mean | 2.68 | 2.77 | 2.20 | 3.76 |
| SD | 2.11 | 2.54 | 2.27 | 2.03 |

Additional file 1: Table S2

The number of ROI and area of ROI (mm^2^) drawn in the dorsal or ventral SAS dependent on detectable CSF flow signal within that region, where 0 corresponds to no CSF flow detected in that region. (*) animals excluded from temporal data analyses and (**) animals excluded from all analyses. ROI = region of interest, CSF = cerebrospinal fluid.

|  | C2/C3 | | | | T8/T9 | | | | | T11/T12 | | | | | L1/L2 | | | |
| --- | --- | --- | --- | --- | --- | --- | --- | --- | --- | --- | --- | --- | --- | --- | --- | --- | --- | --- |
|  | Dorsal ROI | Dorsal Area (mm^2^) | Ventral ROI | Ventral Area (mm^2^) | Dorsal ROI | Dorsal Area (mm^2^) | Ventral ROI | Ventral Area (mm^2^) | Dorsal ROI | | Dorsal Area (mm^2^) | Ventral ROI | Ventral Area (mm^2^) | Dorsal ROI | | Dorsal Area (mm^2^) | Ventral ROI | Ventral Area (mm^2^) |
| P001 | 2 | 10.2 | 1 | 12.6 | 1 | 13.6 | 1 | 11.2 | 0 | | 0 | 1 | 11.2 | 1 | | 6.5 | 1 | 9.2 |
| P002 | 2 | 12.2 | 1 | 4.2 | 0 | 0 | 1 | 7.8 | 0 | | 0 | 1 | 11.2 | 1 | | 5.4 | 1 | 8.8 |
| P003 | 2 | 16.7 | 0 | 0 | 0 | 0 | 0 | 0 | 0 | | 0 | 0 | 0 | 1 | | 15.0 | 1 | 15.7 |
| P004* | 2 | 18.1 | 1 | 15.0 | 1 | 10.9 | 1 | 11.9 | 1 | | 9.9 | 1 | 6.5 | 1 | | 17.4 | 1 | 18.4 |
| P005** | 0 | 0 | 0 | 0 | 0 | 0 | 0 | 0 | 0 | | 0 | 0 | 0 | 0 | | 0 | 0 | 0 |
| P006 | 2 | 25.7 | 1 | 17.9 | 1 | 17.0 | 1 | 18.7 | 1 | | 6.4 | 1 | 19.7 | 1 | | 6.5 | 1 | 11.6 |
| P007 | 2 | 17.0 | 1 | 4.8 | 0 | 0 | 1 | 5.4 | 1 | | 10.4 | 1 | 3.1 | 1 | | 10.2 | 1 | 12.6 |
| P008 | 2 | 12.3 | 1 | 4.8 | 1 | 11.2 | 1 | 9.5 | 1 | | 11.2 | 1 | 13.6 | 1 | | 12.6 | 1 | 13.6 |
| P009 | 2 | 16.5 | 1 | 7.8 | 1 | 5.4 | 1 | 9.5 | 1 | | 9.9 | 1 | 11.2 | 0 | | 0 | 1 | 15.0 |
| P010 | 2 | 14.5 | 0 | 0 | 0 | 0 | 1 | 8.8 | 2 | | 9.9 | 1 | 11.9 | 2 | | 8.2 | 1 | 12.2 |
| P011** | 0 | 0 | 0 | 0 | 0 | 0 | 0 | 0 | 0 | | 0 | 0 | 0 | 0 | | 0 | 0 | 0 |
| P012* | 1 | 5.4 | 0 | 0 | 1 | 4.4 | 1 | 6.8 | 1 | | 11.9 | 1 | 9.9 | 1 | | 16.0 | 1 | 17.0 |
| P013** | 0 | 0 | 0 | 0 | 0 | 0 | 0 | 0 | 0 | | 0 | 0 | 0 | 0 | | 0 | 0 | 0 |
| P014 | 2 | 15.0 | 1 | 13.5 | 1 | 7.5 | 1 | 13.3 | 1 | | 15.3 | 0 | 0 | 1 | | 10.5 | 1 | 8.1 |

Additional file 1: Figure S2

Experimental flow data from P004 and P012 were excluded from temporal analyses and plots due to abnormal cardiac gating. Animal P004 in the dorsal (A) and ventral (B) SAS: the full CSF flow oscillation (diastolic and systolic flow) was not captured in the cycle. Animal P012 in the dorsal (C) and ventral (D) SAS: systolic pulse was acquired at the beginning of the cycle instead of the diastolic pulse. These temporal errors are probably due to incorrect cardiac gating. SAS = subarachnoid space.


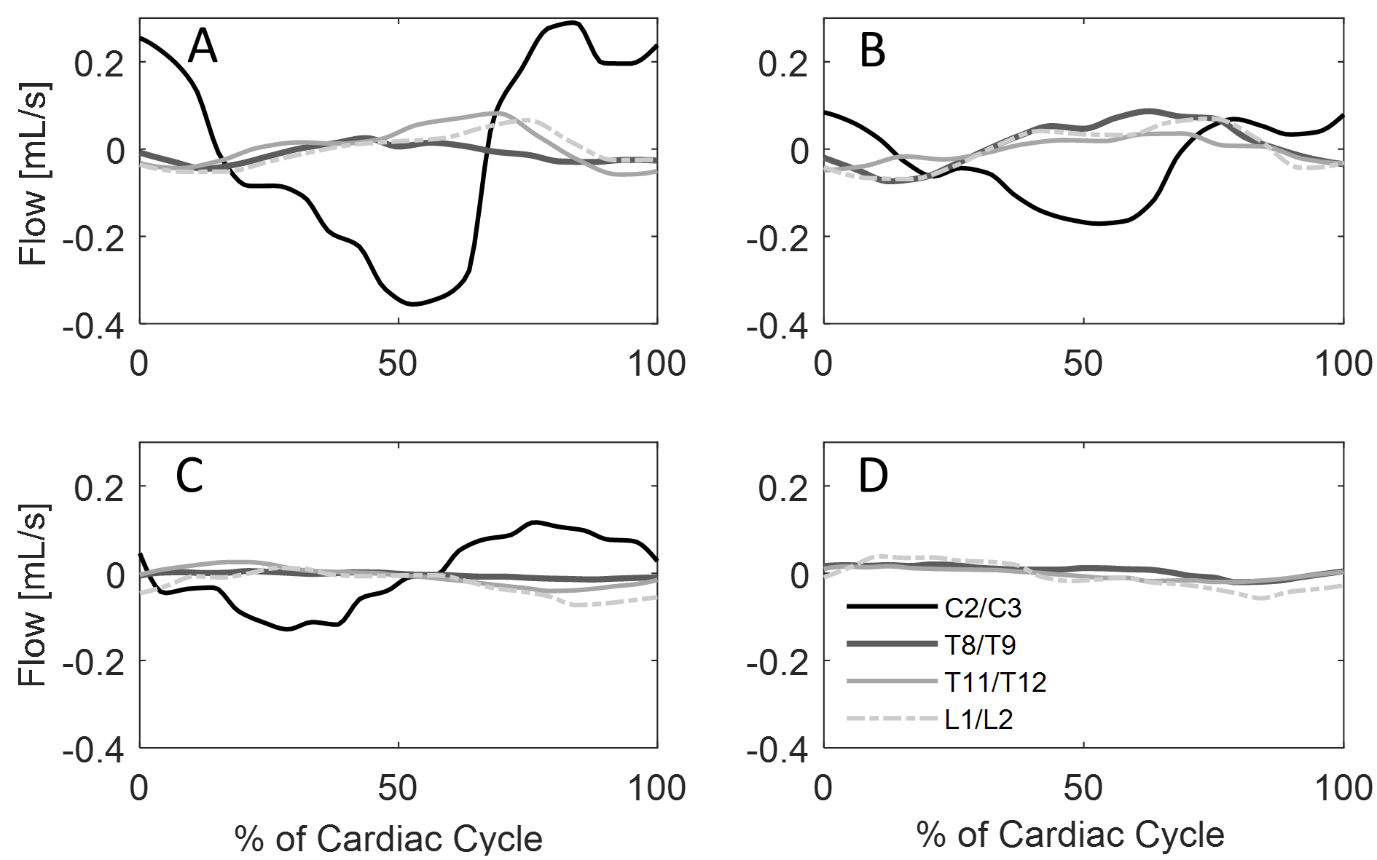


Additional file 1: Table S3

Peak systolic and diastolic flow values (mL/s) for each animal and spinal, with means ± one standard deviation. SAS = subarachnoid space. (*) Animals with abnormal cardiac gating where optimal eddy current corrections could not be performed to achieve zero net flow.

|  | Peak systolic flow (mL/s) in the dorsal SAS | | | | Peak systolic flow (mL/s) in the ventral SAS | | | |
| --- | --- | --- | --- | --- | --- | --- | --- | --- |
|  | **C2/C3** | **T8/T9** | **T11/T2** | **L1/L2** | **C2/C3** | **T8/T9** | **T11/T12** | **L1/L2** |
| P001 | -0.20 | -0.01 | NA | -0.06 | -0.15 | -0.03 | -0.04 | -0.03 |
| P002 | -0.36 | NA | NA | -0.03 | -0.06 | -0.04 | -0.06 | -0.08 |
| P003 | -0.34 | NA | NA | -0.05 | NA | NA | NA | -0.07 |
| P004 | -0.36* | -0.04* | -0.06* | -0.05* | -0.17* | -0.07* | -0.05* | -0.07* |
| P006 | -0.55 | -0.07 | -0.06 | -0.08 | -0.41 | -0.28 | -0.30 | -0.28 |
| P007 | -0.34 | NA | -0.04 | -0.06 | -0.03 | -0.04 | -0.01 | -0.04 |
| P008 | -0.36 | -0.07 | -0.08 | -0.12 | -0.08 | -0.03 | -0.05 | -0.11 |
| P009 | -0.22 | -0.01 | -0.04 | NA | -0.04 | -0.06 | -0.04 | -0.07 |
| P010 | -0.12 | NA | -0.05 | -0.04 | NA | -0.06 | -0.06 | -0.08 |
| P012 | -0.13* | -0.01* | -0.04* | -0.07* | NA | -0.02* | -0.02* | -0.06* |
| P014 | -0.53 | -0.04 | -0.13 | -0.09 | -0.26 | -0.15 | NA | -0.09 |
| Mean | -0.32 | -0.04 | -0.06 | -0.06 | -0.15 | -0.08 | -0.07 | -0.09 |
| SD | 0.14 | 0.02 | 0.03 | 0.03 | 0.12 | 0.08 | 0.08 | 0.06 |
|  | **Peak diastolic flow (mL/s) in the dorsal SAS** | | | | **Peak diastolic flow (mL/s) in the ventral SAS** | | | |
|  | **C2/C3** | **T8/T9** | **T11/T12** | **L1/L2** | **C2/C3** | **T8/T9** | **T11/T12** | **L1/L2** |
| P001 | 0.15 | 0.01 | NA | 0.03 | 0.09 | 0.02 | 0.05 | 0.05 |
| P002 | 0.27 | NA | NA | 0.02 | 0.06 | 0.05 | 0.09 | 0.07 |
| P003 | 0.20 | NA | NA | 0.06 | NA | NA | NA | 0.14 |
| P004 | 0.29* | 0.03* | 0.08* | 0.07* | 0.08* | 0.09* | 0.04* | 0.07* |
| P006 | 0.26 | 0.07 | 0.07 | 0.13 | 0.18 | 0.27 | 0.27 | 0.27 |
| P007 | 0.26 | NA | 0.05 | 0.01 | 0.06 | 0.03 | 0.01 | 0.07 |
| P008 | 0.37 | 0.06 | 0.07 | 0.08 | 0.07 | 0.05 | 0.05 | 0.07 |
| P009 | 0.20 | 0.02 | 0.05 | NA | 0.06 | 0.06 | 0.06 | 0.06 |
| P010 | 0.14 | NA | 0.02 | 0.01 | NA | 0.04 | 0.02 | 0.05 |
| P012 | 0.12* | 0.01* | 0.03* | 0.01* | NA | 0.02* | 0.02* | 0.04* |
| P014 | 0.26 | 0.06 | 0.12 | 0.11 | 0.17 | 0.17 | NA | 0.14 |
| Mean | 0.23 | 0.03 | 0.06 | 0.05 | 0.10 | 0.08 | 0.07 | 0.09 |
| SD | 0.07 | 0.02 | 0.03 | 0.04 | 0.05 | 0.08 | 0.07 | 0.06 |

Additional file 1: Table S4

Physiological recordings and anaesthetic drug levels of each animal during the PC-MRI scan. (*) animals excluded from temporal data analyses, and (**) animals excluded from all analyses. PC-MRI = phase contrast magnetic resonance imaging.

|  | Physiological Recordings | | | | Anaesthetic drug levels | | | |
| --- | --- | --- | --- | --- | --- | --- | --- | --- |
|  | Heart Rate (bpm) | Oxygen Saturation (%) | Respiratory Rate (breaths/min) | End tidal C02 (mmHg) | Propofol at beginning of scan (ml/hr) | Ketamine at beginning of scan  (ml/hr) | Fentanyl at beginning of scan  (ml/hr) | Propofol bolus during scan (ml) |
| P001 | 106 – 118 | 99 | 21 | 52 − 56 | 9 | 1.5 | 3.1 | 0 |
| P002 | 107 − 108 | 96 – 97 | 20 | 41 − 46 | 5 | 0 | 2 | 0 |
| P003 | 74 | 99 | 18 − 19 | 58 | 7.6 | 0 | 2.6 | 0 |
| P004* | 113 – 116^a^ | 98 – 97 | 18 | 50 | 8 | 2.6 | 2.8 | 0 |
| P006 | 73 − 76 | 97 | 18 | 32 | 7.2 | 2.6 | 2.7 | 0 |
| P007 | 80 – 85 | 98 | 18 – 19 | 44 − 50 | 9.5 | 3.4 | 3 | 0 |
| P008 | 96 − 103 | 99 | 19 | 50 | 7.3 | 1 | 2 | 0 |
| P009 | 93 – 104 | 96 | 19 – 20 | 44 – 45 | 7 | 1.8 | 2.5 | 0 |
| P010 | 96 | 100 | 19 – 20 | 46 | 7 | 1.5 | 2.3 | 0 |
| P012* | 174^a^ | 93 | 18 | 47 | 9.9 | 2.3 | 0 | 0 |
| P014 | 71 − 83 | 98 − 99 | 17 | 31 − 45 | 9.8 | 0 | 1.6 | 5 |
| Range | 71 − 174 | 96 – 100 | 17 − 21 | 41 − 58 | 5 − 9.8 | 0 – 3.4 | 2 – 3.1 | - |
| P005** | 94 | 100 | 18 – 19 | 38 | 7.1 | 2.5 | 0 | 0 |
| P011** | 118 – 125 | 98 | 17 | 43 - 46 | 7.5 | 2 | 0 | 0 |
| P013** | 91 | 98 | 18 | 41 | 8.2 | 3 | 2.8 | 0 |
| ^a^Heart rate recorded from pulse oximeter | | | | | | | | |

Additional file 1: Table S5

Stroke volumes (mL/cycle) for each animal and spinal level, with means ± one standard deviation. SAS = subarachnoid space.

|  | Stroke Volume (mL/cycle) | | | | | | | | | | | |
| --- | --- | --- | --- | --- | --- | --- | --- | --- | --- | --- | --- | --- |
|  | **Dorsal SAS** | | | | **Ventral SAS** | | | | **Dorsal + Ventral SAS** | | | |
|  | **C2/C3** | **T8/T9** | **T11/T12** | **L1/L2** | **C2/C3** | **T8/T9** | **T11/T12** | **L1/L2** | **C2/C3** | **T8/T9** | **T11/T12** | **L1/L2** |
| P001 | 0.06 | 0.00 | NA | 0.02 | 0.04 | 0.01 | 0.02 | 0.01 | 0.09 | 0.01 | 0.02 | 0.03 |
| P002 | 0.09 | NA | NA | 0.01 | 0.02 | 0.01 | 0.02 | 0.02 | 0.11 | NA | 0.02 | 0.03 |
| P003 | 0.12 | NA | NA | 0.03 | NA | NA | NA | 0.04 | 0.12 | NA | NA | 0.07 |
| P006 | 0.17 | 0.03 | 0.03 | 0.05 | 0.11 | 0.13 | 0.13 | 0.13 | 0.28 | 0.16 | 0.16 | 0.17 |
| P007 | 0.11 | NA | 0.02 | 0.01 | 0.02 | 0.01 | 0.00 | 0.02 | 0.12 | 0.01 | 0.02 | 0.03 |
| P008 | 0.10 | 0.02 | 0.02 | 0.02 | 0.02 | 0.01 | 0.01 | 0.02 | 0.12 | 0.03 | 0.04 | 0.05 |
| P009 | 0.06 | 0.00 | 0.01 | NA | 0.01 | 0.02 | 0.01 | 0.02 | 0.07 | 0.02 | 0.02 | 0.02 |
| P010 | 0.04 | NA | 0.01 | 0.01 | NA | 0.01 | 0.01 | 0.02 | 0.04 | 0.01 | 0.02 | 0.02 |
| P014 | 0.10 | 0.02 | 0.06 | 0.05 | 0.07 | 0.08 | NA | 0.05 | 0.17 | 0.10 | 0.06 | 0.09 |
| Mean | 0.09 | 0.02 | 0.03 | 0.02 | 0.04 | 0.03 | 0.03 | 0.04 | 0.12 | 0.05 | 0.04 | 0.06 |
| SD | 0.04 | 0.01 | 0.02 | 0.02 | 0.03 | 0.04 | 0.04 | 0.03 | 0.06 | 0.05 | 0.05 | 0.05 |

Additional file 1: Table S6

Estimated marginal means with 95% CI for peak diastolic and systolic flow, and maximum cranial and caudal velocity from LMMs. LMM = linear mixed effects models

| **Spinal level** | **Estimated marginal means for peak diastolic flow (mL/s) with 95% CI** | | **Estimated marginal means for peak systolic flow (mL/s) with 95% CI** | |
| --- | --- | --- | --- | --- |
|  | Dorsal | Ventral | Dorsal | Ventral |
| C2/C3 | 0.23 (0.19, 0.27) | 0.09 (0.05, 0.13) | -0.32 (-0.37, -0.27) | -0.14 (-0.20, -0.08) |
| T8/T9 | 0.03 (-0.02, 0.07) | 0.08 (0.04, 0.12) | -0.03 (-0.09, 0.04) | -0.08 (-0.13, -0.02) |
| T11/T12 | 0.06 (0.02, 0.10) | 0.07 (0.03, 0.11) | -0.06 (-0.11, 0.00) | -0.08 (-0.13, -0.02) |
| L1/L2 | 0.05 (0.01, 0.09) | 0.09 (0.06, 0.13) | -0.06 (-0.11, -0.01) | -0.09 (-0.14, -0.04) |
|  | **Estimated marginal means for maximum cranial velocity (cm/s) with 95% CI** | | **Estimated marginal means for maximum caudal velocity (cm/s) with 95% CI** | |
|  | Dorsal | Ventral | Dorsal | Ventral |
| C2/C3 | 7.04 (5.93, 8.15) | 4.11 (2.88, 5.34) | -7.50 (-8.63, -6.37) | -5.60 (-6.86, -4.35) |
| T8/T9 | 1.49 (0.20, 2.78) | 2.03 (0.89, 3.18) | -1.21 (-2.53, 0.11) | -2.27 (-3.43, -1.10) |
| T11/T12 | 2.81 (1.58, 4.04) | 2.59 (1.41, 3.74) | -2.69 (-3.95, -1.43) | -3.50 (-4.76, -2.23) |
| L1/L2 | 2.61 (1.52, 3.80) | 2.13 (1.02, 3.24) | -2.26 (-3.42, -1.10) | -2.32 (-3.44, -1.19) |

Additional file 1: Table S7

Summary of results from the LMM pairwise comparisons for peak systolic and diastolic flow and maximum cranial and caudal velocity. There was a significant interaction factor between spinal level and SAS region for all four outcome measures. LMM = linear mixed effects models

|  | **Peak diastolic flow (mL/s)** | | **Peak systolic flow (mL/s)** | |
| --- | --- | --- | --- | --- |
| **Dorsal** | **Estimated mean difference and 95% CI** | **p-value** | **Estimated mean difference and 95% CI** | **p-value** |
| C2/C3 vs T8/T9 | 0.20 (0.15, 0.26) | <0.001 | -0.29 (-0.38, -0.21) | <0.001 |
| C2/C3 vs T11/T12 | 0.17 (0.12, 0.22) | <0.001 | -0.27 (-0.35, -0.18) | <0.001 |
| C2/C2 vs L1/L2 | 0.18 (0.13, 0.23) | <0.001 | -0.26 (-0.34, -0.18) | <0.001 |
| T8/T9 vs T11/T12 | -0.03 (-0.10, 0.03) | 0.890 | 0.03 (-0.06, 0.12) | 1.000 |
| T8/T9 vs L1/L2 | -0.03 (-0.08, 0.03) | 1.000 | 0.04 (-0.05, 0.12) | 1.000 |
| T11/T12 vs L1/L2 | -0.01 (-0.05, 0.06) | 1.000 | 0.01 (-0.08, 0.09) | 1.000 |
| **Ventral** | **Estimated mean difference and 95% CI** | **p-value** | **Estimated mean difference and 95% CI** | **p-value** |
| C2/C3 vs T8/T9 | -0.01 (-0.05, 0.06) | 1.000 | -0.06 (-0.14, -0.02) | 0.275 |
| C2/C3 vs T11/T12 | -0.01 (-0.04, 0.07) | 1.000 | -0.06 (-0.15, -0.02) | 0.258 |
| C2/C2 vs L1/L2 | -0.01 (-0.06, 0.05) | 1.000 | -0.05 (-0.13, -0.03) | 0.511 |
| T8/T9 vs T11/T12 | 0.01 (-0.05, 0.06) | 1.000 | -0.00 (-0.08, 0.08) | 1.000 |
| T8/T9 vs L1/L2 | -0.01 (-0.06, 0.04) | 1.000 | 0.01 (-0.07, 0.09) | 1.000 |
| T11/T12 vs L1/L2 | -0.02 (-0.07, 0.03) | 1.000 | 0.01 (-0.07, 0.09) | 0.856 |
|  | **Maximum cranial velocity (cm/s)** | | **Maximum caudal velocity (cm/s)** | |
| **Dorsal** | **Estimated mean difference and 95% CI** | **p-value** | **Estimated mean difference and 95% CI** | **p-value** |
| C2/C3 vs T8/T9 | 5.55 (3.69, 7.41) | <0.001 | -5.74 (-7.60, -3.88) | <0.001 |
| C2/C3 vs T11/T12 | 4.23 (2.45, 6.01) | <0.001 | -4.20 (-5.98, -2.42) | <0.001 |
| C2/C2 vs L1/L2 | 4.38 (2.72, 6.04) | <0.001 | -4.71 (-6.37, -3.05) | <0.001 |
| T8/T9 vs T11/T12 | -1.32 (-3.31, 0.67) | 0.445 | 1.54 (-0.45, 3.53) | 0.230 |
| T8/T9 vs L1/L2 | -1.17 (-3.08, 0.73) | 0.588 | 1.03 (-0.87, 2.94) | 0.861 |
| T11/T12 vs L1/L2 | 0.15 (-1.68, 1.97) | 1.000 | -0.51 (-2.33, 1.32) | 1.000 |
| **Ventral** | **Estimated mean difference and 95% CI** | **p-value** | **Estimated mean difference and 95% CI** | **p-value** |
| C2/C3 vs T8/T9 | 2.08 (0.27, 3.89) | 0.016 | -2.62 (-4.43, -0.82) | <0.001 |
| C2/C3 vs T11/T12 | 1.52 (-0.34, 3.38) | 0.178 | -1.58 (-3.44, -0.29) | 0.146 |
| C2/C2 vs L1/L2 | 1.98 (0.20, 3.76) | 0.022 | -2.61 (-4.39, -0.82) | <0.001 |
| T8/T9 vs T11/T12 | -0.56 (-2.30, 1.19) | 1.000 | -1.04 (-0.71, 2.79) | 0.651 |
| T8/T9 vs L1/L2 | -0.10 (-1.76, 1.57) | 1.000 | 0.01 (-1.65, 1.68) | 1.000 |
| T11/T12 vs L1/L2 | 0.46 (-1.26, 2.18) | 1.000 | -1.03 (-2.75, 0.69) | 0.641 |

Additional file 1: Figure S3

Mean CSF velocity in the dorsal and ventral region of the SAS at each spinal level. Data presented as means ± one standard deviation as shaded area. CSF = cerebrospinal fluid, SAS = subarachnoid space.


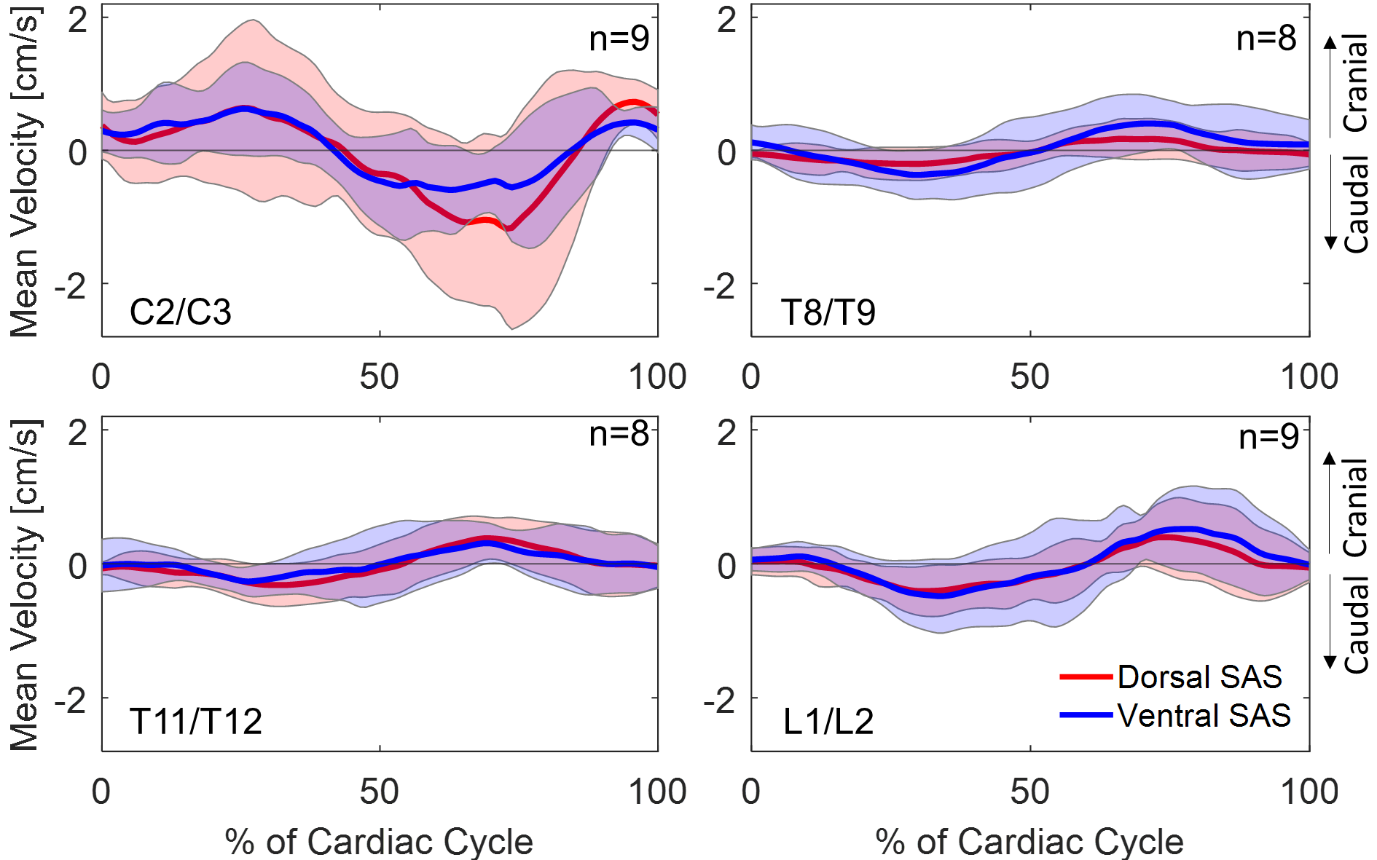


Additional file 1: Table S8

Time to peak systolic and diastolic velocity (as a percentage of the cardiac cycle) and cardiac cycle duration (ms) for each animal and spinal level. SAS = subarachnoid space.

|  | Time to peak systolic velocity in the dorsal SAS (% of cardiac cycle) | | | | Time to peak systolic velocity in the ventral SAS (% of cardiac cycle) | | | |
| --- | --- | --- | --- | --- | --- | --- | --- | --- |
|  | **C2/C3** | **T8/T9** | **T11/T2** | **L1/L2** | **C2/C3** | **T8/T9** | **T11/T12** | **L1/L2** |
| P001 | 64.7 | 08.1 | NA | 20.2 | 55.6 | 08.1 | NA | 20.2 |
| P002 | 58.6 | NA | NA | 28.3 | 52.5 | 25.3 | 25.3 | 29.3 |
| P003 | 80.8 | NA | NA | 28.3 | NA | NA | NA | 24.2 |
| P006 | 74.8 | 43.4 | 48.5 | 32.3 | 80.8 | 47.5 | 51.5 | 54.6 |
| P007 | 70.7 | NA | 41.4 | 47.5 | 59.6 | 36.4 | 45.5 | 52.5 |
| P008 | 63.6 | 29.3 | 24.2 | 33.3 | 63.6 | 29.3 | 23.2 | 33.3 |
| P009 | 72.7 | 26.3 | 26.3 | NA | 67.7 | 26.3 | 26.3 | 45.5 |
| P010 | 70.7 | NA | 33.3 | 33.3 | NA | 29.3 | 28.2 | 28.3 |
| P014 | 73.7 | 12.1 | 37.4 | 45.5 | 73.7 | 29.3 | NA | 33.3 |
|  | **Time to peak diastolic in the dorsal SAS (% of cardiac cycle)** | | | | **Time to peak diastolic in the ventral SAS (% of cardiac cycle)** | | | |
|  | **C2/C3** | **T8/T9** | **T11/T12** | **L1/L2** | **C2/C3** | **T8/T9** | **T11/T12** | **L1/L2** |
| P001 | 18.2 | 53.5 | NA | 66.7 | 10.1 | 53.5 | 56.6 | 72.7 |
| P002 | 18.2 | NA | NA | 71.7 | 12.1 | 68.7 | 56.6 | 77.8 |
| P003 | 43.4 | NA | NA | 75.8 | NA | NA | NA | 85.9 |
| P006 | 33.3 | 86.9 | 77.8 | 77.8 | NA | 86.9 | 86.9 | 85.9 |
| P007 | 37.4 | NA | 68.7 | 100.0 | 26.3 | 72.7 | 72.7 | 89.9 |
| P008 | 23.2 | 59.6 | 70.7 | 73.7 | 27.2 | 64.7 | 64.7 | 72.7 |
| P009 | 31.3 | 68.7 | 63.6 | NA | 31.3 | 68.7 | 63.6 | 84.9 |
| P010 | 33.3 | NA | 66.7 | 66.7 | NA | 81.8 | 83.8 | 83.8 |
| P014 | 16.2 | 70.7 | 66.7 | 66.7 | 16.2 | 70.7 | NA | 66.7 |
|  | **Cardiac cycle duration (ms)** | | | |  |  |  |  |
|  | **C2/C3** | **T8/T9** | **T11/T2** | **L1/L2** |  |  |  |  |
| P001 | 561.0 | 526.4 | 522.7 | 506.7 |  |  |  |  |
| P002 | 550.6 | 558.1 | 555.3 | 551.5 |  |  |  |  |
| P003 | 805.3 | 805.3 | 805.3 | 804.1 |  |  |  |  |
| P006 | 817.6 | 812.7 | 800.2 | 784.9 |  |  |  |  |
| P007 | 735.5 | 748.0 | 734.6 | 701.3 |  |  |  |  |
| P008 | 624.0 | 608.2 | 592.2 | 578.0 |  |  |  |  |
| P009 | 642.2 | 625.1 | 609.0 | 573.3 |  |  |  |  |
| P010 | 619.2 | 623.3 | 624.3 | 624.3 |  |  |  |  |
| P014 | 718.1 | 742.1 | 819.8 | 843.8 |  |  |  |  |

Additional file 1: Table S9

Peak mean systolic and diastolic velocity values (mL/s) for each animal and spinal level, with mean ± one standard deviation. SAS = subarachnoid space. (*) Animals with abnormal cardiac gating where optimal eddy current corrections could not be performed to achieve zero net flow.

|  | Peak mean systolic velocity (cm/s) in the dorsal SAS | | | | Peak mean systolic velocity (cm/s) in the ventral SAS | | | |
| --- | --- | --- | --- | --- | --- | --- | --- | --- |
|  | **C2/C3** | **T8/T9** | **T11/T2** | **L1/L2** | **C2/C3** | **T8/T9** | **T11/T12** | **L1/L2** |
| P001 | -1.76 | -0.09 | NA | -0.37 | -1.17 | -0.26 | -0.34 | -0.20 |
| P002 | -3.01 | NA | NA | -0.47 | -1.43 | -0.48 | -0.50 | -0.89 |
| P003 | -2.05 | NA | NA | -0.35 | NA | NA | NA | -0.47 |
| P004 | -2.01* | -0.38* | -0.49* | -0.30* | -1.14* | -0.54* | -0.89* | -0.37* |
| P006 | -2.16 | -0.42 | -0.97 | -1.24 | -2.30 | -1.49 | -1.51 | -2.39 |
| P007 | -1.99 | NA | -0.38 | -0.54 | -0.61 | -0.79 | -0.48 | -0.30 |
| P008 | -2.95 | -0.61 | -0.73 | -0.94 | -1.71 | -0.37 | -0.34 | -0.80 |
| P009 | -1.31 | -0.23 | -0.39 | NA | -0.47 | -0.63 | -0.34 | -0.44 |
| P010 | -0.92 | NA | -0.46 | -0.43 | NA | -0.69 | -0.51 | -0.66 |
| P012 | -2.39* | -0.32* | -0.34* | -0.46* | NA | -0.30* | -0.20* | -0.34* |
| P014 | -3.56 | -0.55 | -0.82 | -0.87 | -1.80 | -1.11 | NA | -1.14 |
| Mean | -2.19 | -0.37 | -0.57 | -0.60 | -1.33 | -0.67 | -0.57 | -0.73 |
| SD | 0.73 | 0.17 | 0.22 | 0.30 | 0.57 | 0.37 | 0.38 | 0.59 |
|  | **Peak mean diastolic velocity (cm/s) in the dorsal SAS** | | | | **Peak mean diastolic velocity (cm/s) in the ventral SAS** | | | |
|  | **C2/C3** | **T8/T9** | **T11/T12** | **L1/L2** | **C2/C3** | **T8/T9** | **T11/T12** | **L1/L2** |
| P001 | 1.23 | 0.09 | NA | 0.19 | 0.73 | 0.17 | 0.42 | 0.34 |
| P002 | 2.07 | NA | NA | 0.40 | 1.51 | 0.64 | 0.80 | 0.83 |
| P003 | 1.19 | NA | NA | 0.42 | NA | NA | NA | 0.88 |
| P004 | 1.64* | 0.21* | 0.69* | 0.38* | 0.56* | 0.64* | 0.65* | 0.38* |
| P006 | 1.00 | 0.42 | 1.10 | 1.95 | 1.00 | 1.44 | 1.35 | 2.34 |
| P007 | 1.40 | NA | 0.48 | 0.10 | 1.21 | 0.57 | 0.46 | 0.50 |
| P008 | 3.04 | 0.50 | 0.64 | 0.64 | 1.51 | 0.57 | 0.35 | 0.51 |
| P009 | 1.17 | 0.29 | 0.53 | NA | 0.75 | 0.65 | 0.52 | 0.42 |
| P010 | 1.10 | NA | 0.19 | 0.16 | NA | 0.42 | 0.20 | 0.38 |
| P012 | 2.16* | 0.11* | 0.22* | 0.07* | NA | 0.30* | 0.17* | 0.23* |
| P014 | 1.71 | 0.77 | 0.76 | 1.09 | 1.13 | 1.29 | NA | 1.69 |
| Mean | 1.61 | 0.34 | 0.58 | 0.54 | 1.05 | 0.67 | 0.55 | 0.77 |
| SD | 0.58 | 0.22 | 0.28 | 0.55 | 0.33 | 0.38 | 0.34 | 0.63 |
